# Emerging host-directed strategies for overcoming drug resistance and immune evasion in *Staphylococcus aureus* infections

**DOI:** 10.1016/j.jare.2025.08.001

**Published:** 2025-08-05

**Authors:** Youle Zheng, Jin Feng, Qianwei Qu, Yongzheng Liu, Yadan Zheng, Yanhua Li

**Affiliations:** a College of Veterinary Medicine, Northeast Agricultural University, Harbin 150030, China; b Heilongjiang Key Laboratory for Animal Disease Control and Pharmaceutical Development, Harbin 150030, China; c Key Laboratory of the Provincial Education Department of Heilongjiang for Common Animal Disease Prevention and Treatment, Northeast Agricultural University, Harbin 150030, China

**Keywords:** Host-directed therapy, *Staphylococcus aureus*, Intracellular infection, Immune regulation, Drug-target interaction

## Abstract

**Background:**

*Staphylococcus aureus* poses a significant global threat to both human and animal health. Bacterial-targeted therapies are limited by strong selective pressures (e.g., *mecA*-mediated β-lactam resistance) and the pathogen’s remarkable capacity for phagocyte intracellular survival—a strategy that allows them to evade antibiotics and the immune system, ultimately leading to persistent infections. These factors highlight the urgent need for alternative strategies to combat bacterial infections.

**Aim of review:**

This review explores host-directed therapies (HDTs) as alternative strategies for combating *S. aureus* infections, aiming to overcome limitations of current treatments and contribute to the development of novel or combination therapies.

**Key scientific concepts of review:**

HDTs offer several advantages, including a reduced risk of bacterial resistance, a broad antimicrobial spectrum, and the potential for synergy with antibiotics to shorten treatment duration and even treat persistent intracellular infections. Target-based precision drug design shows strong promise in addressing off-target effect challenges. This review provides an overview of key host factors involved in *S. aureus* pathogenesis, emphasizing therapeutic agents targeting these factors, their mechanisms of action, and pharmacological profiles. These recent advancements are crucial in the ongoing efforts to combat *S. aureus* infections.

## Highlights

• Traditional anti-*S. aureus* therapies face challenges from resistant strains.
• Host-directed therapies (HDTs) target host immune, inflammatory, and other pathways.
• HDTs show potential in overcoming drug resistance and immune evasion in infections.
• Host targets (e.g., AMPK, dCK, PDE4) are promising for anti-*S. aureus* drug design.

## Graphical abstract

This review examines the role of host factors in *S. aureus* infection and categorizes potential HDADs, analyzing the interactions between these drugs and key host factors.

**Figure:**
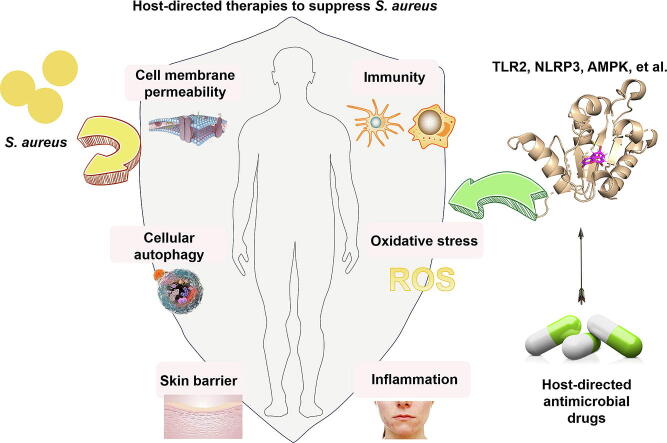

## Introduction

*Staphylococcus aureus* is a facultative intracellular pathogen that is linked to a wide range of severe health conditions [1], such as skin and soft tissue infections [2], endocarditis [3], septic arthritis [4], osteomyelitis [5], and systemic issues like bacteremia and sepsis [6,7]. The prevention and treatment of *S. aureus* infections in clinical practice primarily rely on antibiotic therapies. However, the extensive use of antibiotics has exerted significant selective pressure, driving the emergence of multidrug-resistant strains like methicillin-resistant *S. aureus* (MRSA), which now poses an escalating threat to public health worldwide [8]. The mortality rate associated with this pathogen ranges from 10 % to 60 % [[9], [10], [11], [12], [13]], with deaths now exceeding those caused by acquired immunodeficiency syndrome, tuberculosis, and viral hepatitis [14,15]. The development of new antibiotics is both high-risk and expensive, with a success rate of less than 12 % from phase I to market and total costs exceeding $1 billion [16]. Furthermore, antibiotics that target microbes are prone to resistance, thereby diminishing their effectiveness. The discovery and development of new antibiotics has become increasingly unprofitable, jeopardizing our capacity to control infections [17]. These challenges highlight the need for innovative approaches like host-directed therapies (HDTs), which target host-pathogen interactions rather than microbial components [18].Table 1.

**Table 1:** Agents targeting the host and their mode of action.

Target	Agent	Strain	Note	Ref.
AMPK	Dorsomorphin	USA300 LAC and NCTC 13626	By inhibiting AMPK, it disrupts intracellular MRSA's ability to utilize autophagy for nutrient scavenging and energy production	[152]
AMPK	GW296115X	USA300 LAC JE2, USA300 BK 11540, and LUH 15392	It activates AMPK, thereby enhancing bacterial degradation *via* autophagy and improving the survival rates of MRSA-infected zebrafish embryos	[153]
Caspase	Emricasan	USA300 LAC::*lux*	It reduces lesion size and bacterial burden in *S. aureus* skin infections by targeting host cell apoptosis pathways, rather than directly acting as an antibacterial agent	[97]
Caspase	Q-VD-OPH	USA300 LAC::*lux*	It effectively treats *S. aureus* skin infections in mice by inhibiting caspases-3, −8, and −9	[98]
CD44	Hyaluronic acid-streptomycin-lipoic acid-glabrol nanoparticles	USA 300, MRSA 011, MRSA 003, MRSA 031, ATCC 25923, and ATCC 43300	It targets infected macrophages *via* CD44, delivers glabrol-streptomycin with glutathione-triggered release	[113]
Deoxycytidine kinase	(R)-DI-87	Newman wild type and Newman Δ*adsA*	By suppressing bacterial-induced macrophage cell death, it mitigates *S. aureus* abscess formation in organ tissues during invasive bloodstream infection	[90]
EGFR-STAT1/3 pathway	DRGN-1	SH1000	It enhances wound healing by promoting keratinocyte migration and activating the EGFR-STAT1/3 signaling pathway	[147]
ERK, JNK, MAPK, and NF-κB pathways	Baicalin	HS488	It inhibits pro-inflammatory cytokine production in MRSA infections, reducing the high mortality rate in mice	[137]
FPR2	WKYMVm	USA300	It is a synthetic hexapeptide that can stimulate human monocytes to produce superoxide and kill *S. aureus*	[83]
GAPDH	4-Octyl itaconate	USA300 LAC	It has anti-inflammatory effects by targeting GAPDH to decrease aerobic glycolysis in macrophages	[218]
HCAR2	Nicotinic acid	*S. aureus*	It exerts antimicrobial effects in mouse mammary infections by activating HCAR2 and preventing NLRP3 inflammasome activation	[140]
Histone deacetylase 3	High-inulin	ATCC 35,556	It enhances the host's antimicrobial program in macrophages by inhibiting histone deacetylase 3, providing therapeutic effects against *S. aureus*-induced mastitis in mice	[219]
HMG-CoA reductase	Statins	*S. aureus*	They inhibit the production of prenylation intermediates, disrupting *S. aureus*' ability to invade host cells	[156]
Lysosomes	2-deoxyglucose	RN6390	It reduces intraocular inflammation in *S. aureus*-infected mouse eyes through the inhibition of the MEK/ERK pathway	[95]
Lysosomes	OG9	ATCC 25,904	It targets and disrupts both the bacterial membranes and DNA within the phagolysosome, overcoming antibiotic resistance mechanisms associated with bacterial dormancy and subcellular sequestration	[93]
Lysosomes	TTTh	*S. aureus* 29,213 and MRSA	It targets lysosomes to enhance their maturation, induces ROS accumulation, and clears bacterial burden in the wound	[32]
Macrophages	Cip-CBT-Ada	*S. aureus*	It targets macrophages to deliver ciprofloxacin nanoparticles, showing excellent intracellular bacterial elimination and inflammation relief in cell and mouse infection models	[110]
Macrophages	CRV/LNP-RNAs	MRSA	It enhances the immune response by delivering CAR mRNA and caspases-11 siRNA to macrophages	[109]
Macrophages	NK cell mimics (loading perforin and granzyme B into modified mesoporous silica nanoparticles)	ATCC 43,300	Perforin forms pores in infected cell membranes, enabling granzyme B to enter and kill pathogens	[118]
Macrophages	Ras-selective lethal small molecule 3, sulfasalazine, and acetaminophen	ATCC 29,213	It enhances ferroptotic stress in macrophages, promoting the delivery of ferrous iron to intracellular bacteria *via* ferroportin, and inducing ferroptosis-like death in the bacteria	[103]
Macrophages	TPE-Parg	ATCC 6538	It stimulates macrophages to produce nitric oxide, activating the immune system to clear *S. aureus* in a subcutaneous mouse infection model	[220]
Macrophages and neutrophils	Gy-CATH	*S. aureus* and MRSA	It is identified from the skin of the frog and prevents bacterial infections in mice by inhibiting thrombosis and regulating neutrophils and macrophages involved in host immune defense	[86]
Macrophages and neutrophils	LL-37	*S. aureus*	It is from the human cathelicidin family and enhances NET formation to physically trap *S. aureus*	[84]
Macrophages and neutrophils	Nv-CATH	*S. aureus*	It is identified from the skin of the frog and combats infection through immune modulation and reducing inflammation	[87]
NLRP3	1-peptidyl-2-arachidonoyl-3-stearoyl-*sn*-glyderide	MRSA	It targets the NLRP3 inflammasome to reduce pathogen load, and outperform antibiotics in treating antibiotic-resistant and emerging infections	[109]
NLRP3	MCC950	USA300 LAC, USA300 LAC Δ*hla*, HG003, and HG003 Δ*hla*	It enhances *in vivo* antibiotic efficacy by inhibiting NLRP3 inflammasome activation	[123]
NLRP3	OLT1177	USA300 FPR3757	It reduces IL-1β production in MRSA-infected synovial tissues by inhibiting NLRP3 activation, thereby protecting joint cartilage from damage in a septic arthritis mouse model	[124]
NOD2-RIP2 signaling axis	PALA	*S. aureus* Reynolds (CP5) and MRSA strain MW2 (SAP227)	It alleviates NOD2 repression in intestinal epithelial cells, boosting bacterial clearance and offering potential against antibiotic-resistant infections in skin wounds	[91]
P2Y12 receptor	Ticagrelor	MRSA and MSSA	Ticagrelor inhibits *S. aureus* by enhancing platelet-mediated killing, preventing α-toxin-related desialylation, and blocking bacterial binding to endothelial tissues	[99]
Phosphodiesterase 4	Crisaborole	USA300 LAC::*lux* and LAC4303	It reduces pruritus in psoriasis-like dermatitis and *S. aureus* skin colonization in an AD-like mouse model	[125]
TLR2	Brazilin	*S. aureus*	It targets TLR2 to modulate the inflammatory response in *S. aureus*-induced mastitis by inhibiting the expression of proinflammatory cytokines and regulating NF-κB and MAPK signaling pathways	[139]
TLR2/MAPKs/NLRP3 signaling pathway	Combination of insulin and linezolid	ATCC 29,213 and 8325–4	They exhibit antibacterial, glucose-lowering, and anti-inflammatory effects *via* the TLR2/MAPKs/NLRP3 pathway in a mouse model of diabetes complicated by *S. aureus* infection	[135]
TLRs	Keratin 6a-derived antimicrobial peptides	*S. aureus*	They alleviate bacterial keratitis by reducing cell surface availability of TLR2 and TLR4 through promotion of receptor endocytosis	[133]
TNFR2	NewSTAR2	USA300 LAC::*lux*	It is a fusion polypeptide derived from mice and shows therapeutic efficacy against *S. aureus* skin infections by enhancing neutrophil extracellular trap formation	[89]
Tyrosine kinase	Ibrutinib, dasatinib, crizotinib	USA300 LAC and USA300-GFP	They inhibit host cell invasion and bacterial proliferation, likely through modulation of host factors such as EPHA2, C-JUN, and NWASP	[33]
TRAM2 and SERCA	Thapsigargin	*S. aureus* NCTC 13626, USA300 LAC, and USA300-GFP	It inhibits the SERCA pumps, disrupting intracellular calcium homeostasis	[157]
Vitamin D receptor	Vitamin D	*S. aureus* strain A1 isolated from cows with mastitis	It inhibits *S. aureus* invasion by reducing cell viability and regulating vitamin D receptor-related gene expression	[96]

Several antibiotic adjuvants and alternative therapies have been proposed [[19], [20], [21], [22], [23], [24]]; however, *S. aureus* persists intracellularly through nutrient exploitation and immune evasion, developing dual resistance to both antibiotics and host defenses [25,26]. Specifically, it employs the two-component system to facilitate bacterial aggregation, effectively concealing pathogen-associated molecular patterns (PAMPs) from immune detection. By evading Toll-like receptor (TLR) 2 recognition, this strategy may suppress nuclear factor-kappa B (NF-κB) activation in macrophages, ultimately impairing critical immune responses such as microbial killing, phagocytic efficiency, reactive oxygen species (ROS) generation, and cytokine release [27]. To evade the complement system, chemotaxis inhibitory protein of *S. aureus* and staphylococcal complement inhibitor A interfere with complement activation by inactivating complement components and blocking immune cell chemotaxis, thus reducing immune cell recruitment to the infection site [28]. Recent studies further reveal that *S. aureus* subverts host defenses by hijacking the caspase-8 signaling pathway, thereby promoting cell survival and suppressing xenophagy [29]. This intracellular survival strategy significantly impedes effective bacterial clearance *in vivo*, particularly when therapeutic approaches target only the bacteria themselves [30,31].

Building on this concept, HDTs offer a promising approach in the fight against antimicrobial resistance and persistent bacterial infections [31,32]. This strategy focuses on targeting host immune and inflammatory pathways, or host cell factors that pathogens exploit for survival, replication, or persistence [33]. By enhancing the host's ability to combat new infections and simultaneously minimizing selective pressure, HDTs reduce the risk of resistance development [31]. Current reviews predominantly focus on antimicrobial strategies targeting bacterial factors, with limited attention given to the summary and future prospects of HDTs. In recent years, there has been a growing shift of focus from bacteria to the host [[34], [35], [36], [37]]. This review explores key host factors and their corresponding therapeutic agents, providing a systematic overview of their classification, mechanisms of action, and pharmacological profiles. These insights are crucial for the development of alternative or combination therapies to manage clinical infections and advance novel antibacterial agents.

## Diseases associated with *S. aureus* and their pathogenesis

**Fig. 1:**
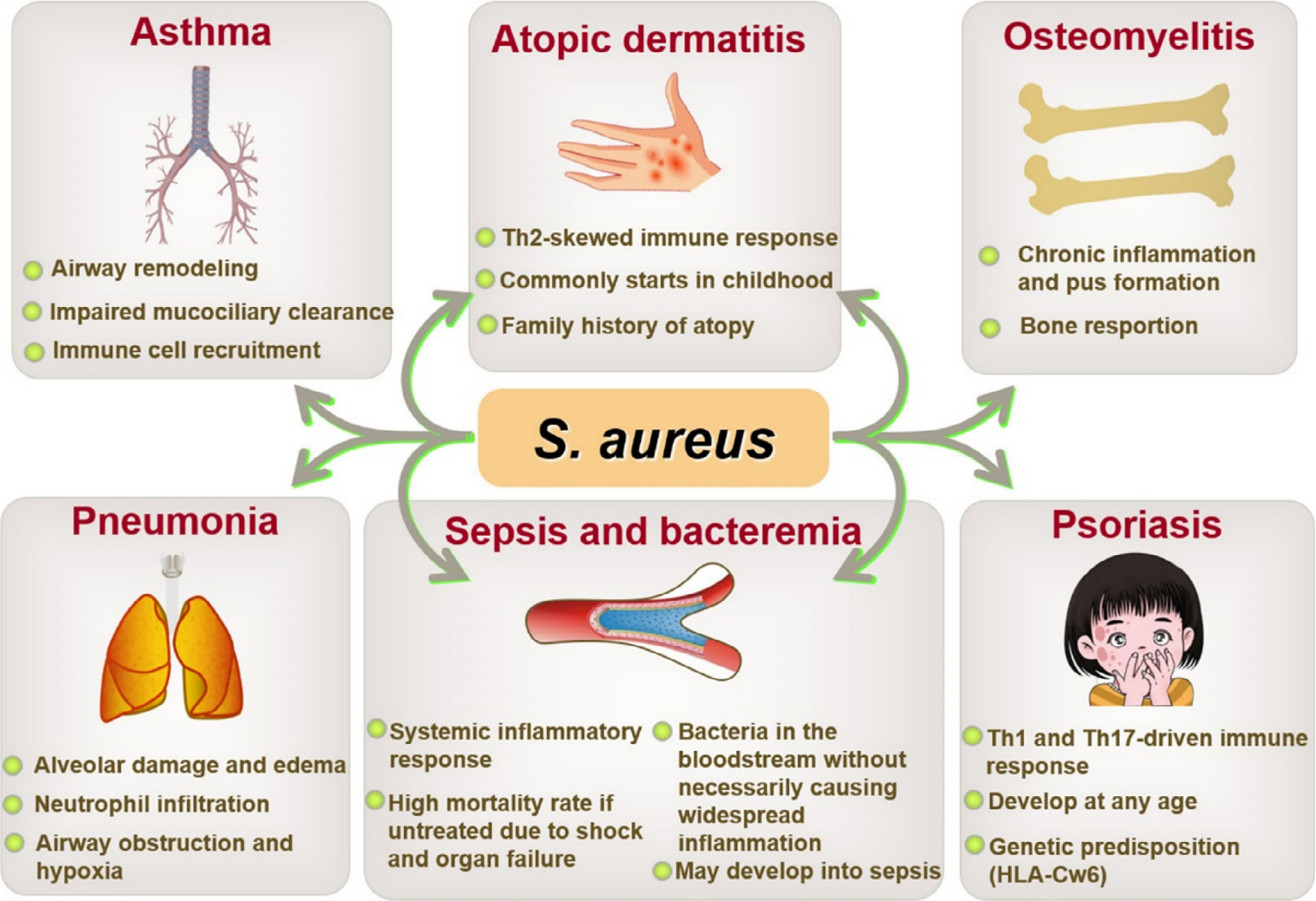
Diseases associated with *S. aureus* and their pathogenesis.

**Fig. 2:**
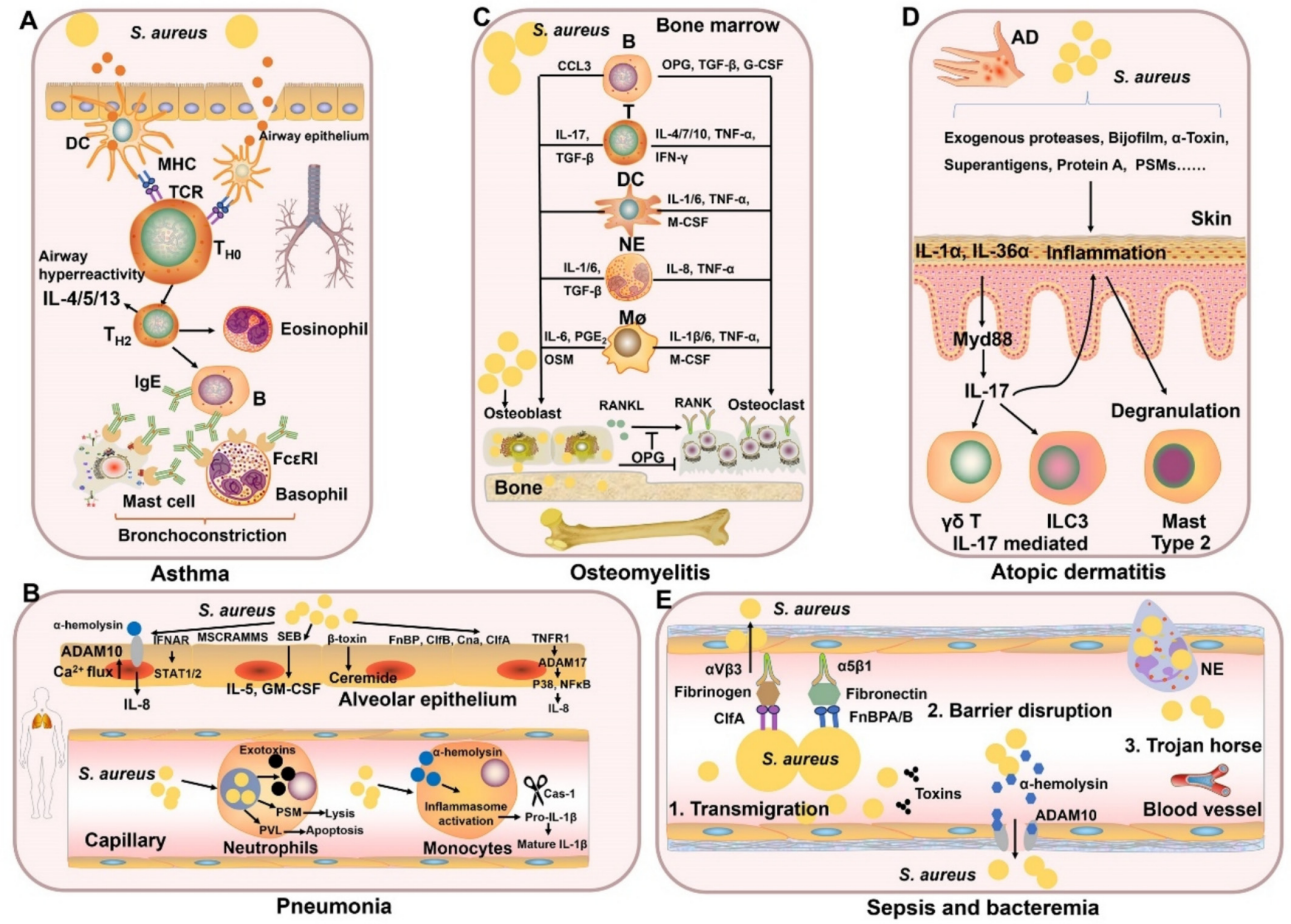
Pathogenesis and signaling pathways in response to *S. aureus*. **A. Models for asthma caused by *S. aureus*.***S. aureus* exotoxins bind to MHC class II molecules on DCs, activating polyclonal T cells and causing excessive cytokine release, including IL-4, IL-5, and IL-13. These cytokines drive eosinophilic inflammation in severe asthma and CRSwNP, with IL-5 mediating the eosinophilic response, and IL-4 and IL-13 promoting B cell activation and IgE class switching. It remains unclear whether locally produced IgE is biologically active and capable of triggering mast cell and basophil activation *via* FcƐ receptors. **B. Models for pneumonia caused by *S. aureus*.***S. aureus* activates IFN signaling in the alveolar epithelium *via* IFNAR1 and STAT1/2. The α-Hemolysin binds ADAM10, inducing calcium influx and releasing inflammatory factors. SEB triggers G/M−CSF release, upregulating IL-5R and promoting eosinophil differentiation. β-Toxin hydrolyzes sphingolipids to ceramide, impairing the endothelial barrier. TNFR1 activation induces IL-8 release. *S. aureus* survives intracellularly in neutrophils, escaping vacuoles *via* exotoxins like PVL and PSMs, which induce neutrophil lysis. In monocytes, α-hemolysin activates the inflammasome, releasing IL-1β. **C. Models for osteomyelitis caused by *S. aureus*.** Immune cells are activated to secrete a variety of cytokines (including IL-1, IL-1β, IL-4, IL-6, IL-7, IL-8, IL-10, and IL-17), growth factors (such as G-CSF and M−CSF), chemokines (like CCL3), and other inflammatory molecules (including TNF-α, TGFβ, IFN-γ, OSM, and PGE2). These factors significantly influence the function of osteoblasts and osteoclasts, thereby modulating bone remodeling. Osteoblasts release OPG, a decoy receptor that binds to RANKL, preventing the RANK-RANKL interaction and thereby inhibiting bone resorption and osteoclast formation. **D. Models for atopic dermatitis caused by *S. aureus*.** Virulence factors disrupt the epithelial barrier, promoting bacterial colonization and activating quorum sensing. This triggers IL-1α and IL-36α release from keratinocytes, initiating IL-17-mediated dermatitis, mast cell degranulation, and Th2-driven inflammation, all of which worsen skin barrier damage and inflammation. **E. Models for bloodstream diseases caused by *S. aureus*.** ClfA binds to fibrinogen, which serves as a ligand for the host's αVβ3 integrin, mediating bacterial adherence. Additionally, FnBPA and FnBPB interact with fibronectin and engage with the α5β1 integrin on the vascular endothelial surface, initiating cellular invasion and facilitating transmigration. The α-hemolysin targets the ADAM10, leading to the disruption of the normal barrier function of the vascular endothelium. Neutrophils transport *S. aureus* into host tissues through a Trojan horse mechanism.

*S. aureus* can cause a wide range of infections, including respiratory system diseases, osteomyelitis, skin disorders, and bloodstream diseases (Fig. 1), with significant clinical challenges due to its ability to colonize tissues, secrete potent toxins, and develop antibiotic resistance. In the respiratory system, *S. aureus* exacerbates conditions such as asthma, chronic rhinosinusitis, and pneumonia. These infections can trigger a type 2 inflammatory response, involving eosinophils and related cytokines [38]. Asthma is a condition that affects millions globally [39]. Various types of T cells, including T helper 2 (Th2) cells, T regulatory cells, and innate lymphoid cells, play critical roles in the host’s response to *S. aureus* and contribute to the development of asthma (Fig. 2A). People with asthma are at increased risk of pneumonia [40]. Various virulence factors can trigger host signaling pathways in airway epithelial cells during pneumonia, including the signal transducer and activator of NF-κB pathways [41]. Additionally, β-toxin generates ceramide, a sphingolipid metabolite, through the hydrolysis of sphingomyelin [42]. This ceramide promotes endothelial dysfunction, inhibits angiogenesis, disrupts the endothelial barrier, and induces senescence, oxidative stress, and cell death [43]. *S. aureus* can also evade the immune system by modulating host immune cells. In monocytes, interaction with *S. aureus* and the action of α-hemolysin lead to the activation of the inflammasome [44]. Moreover, *S. aureus* can persist within neutrophils for several days [45], subsequently escaping vacuolar compartments *via* Panton-Valentine leucocidin (PVL) and phenol-soluble modulins (PSMs), as these exotoxins can lyse neutrophils (Fig. 2B).

Osteomyelitis is an inflammatory bone disease, primarily caused by *S. aureus* and *Staphylococcus epidermidis* infections [46]. Individuals over 40, especially those with diabetes, may develop osteomyelitis due to foot ulcers from neuropathy and vascular insufficiency [47]. The infection typically affects the calcaneus, metatarsals, and toes. Studies report that 12 % to 20 % of diabetic foot ulcer patients develop bone infections, with rates exceeding 66 % in severe cases [46,48,49]. Osteomyelitis remains a significant clinical challenge, with approximately 40 % of patients experiencing recurrent and persistent infections [46]. Bacteria infiltrate the cortical bone and infect osteocytes, causing the infected osteocytes to secrete more chemokines and inflammatory factors, which can promote inflammation and bone destruction (Fig. 2C). Furthermore, bacterial virulence factors and biofilm formation alter the immune environment, leading to immune escape and enhanced bacterial colonization [50]. Antibiotic resistance mechanisms in staphylococci allow them to evade both the host immune response and antibiotic treatments, while surgical interventions aimed at removing necrotic and infected bone can further aggravate the patient's condition.

Skin infections caused by *S. aureus* are common and often complicate various dermatological conditions, including atopic dermatitis (AD). This chronic inflammatory skin disorder affects approximately 10 % to 20 % of children and 7 % to 10 % of adults globally, with a current global prevalence estimated at 204 million people [51]. Clinical symptoms include erythema, pustules, crusting, and the formation of abscesses. *S. aureus* infection may contribute to increased antibiotic use and prolonged disease duration, further complicating treatment and management. Therefore, a comprehensive treatment strategy is essential to mitigate the development of antibiotic resistance [52]. Research indicates that infections are driven by several host factors, such as a compromised skin barrier, immune dysregulation, and skin damage from scratching, which exacerbates pruritus and promotes further scratching, creating a vicious cycle that worsens the skin damage and the risk of secondary infection [53]. Moreover, this colonization further activates the quorum sensing system, promotes the release of interleukin (IL)-1α and IL-36α alarmins from keratinocytes, and initiates IL-17-mediated dermatitis. Additionally, it induces mast cell degranulation and triggers Th2-mediated inflammation (Fig. 2D). These processes further disrupt the skin barrier and exacerbate inflammation, contributing to the recurrence of eczema and intense itching [54]. A *meta*-analysis of 61 studies on 4,091 *S. aureus* isolates from AD patients found that 4 of 11 commonly used antibiotics had susceptibility rates of 85 % or less, indicating suboptimal effectiveness of β-lactams, erythromycin, clindamycin, and fusidic acid for empirical treatment in AD patients [55]. Therefore, targeting host modulation, including immune regulation, inflammation control, and skin barrier enhancement, is crucial for managing *S. aureus* infection in AD and preventing complications [56].

*S. aureus* is a frequent pathogen responsible for serious bloodstream infections [11,57,58]. Once *S. aureus* enters the bloodstream, it triggers a complex cascade of events that undermine the body's normal physiological functions. It has evolved an array of strategies to evade the host's innate immune responses. For instance, it secretes factors like staphylococcal superantigen-like 5 and 10, chemotaxis inhibitory protein, and formyl peptide receptor-like inhibitory proteins to impede neutrophil chemotaxis, reducing immune cell influx to the infection site [59]. Additionally, it secretes proteins such as staphylococcal complement inhibitor and aureolysin to interfere with the complement system, diminishing opsonization essential for phagocytosis. The bacterium also manipulates the hemostatic system. By secreting coagulases like Coa and vWbp, it activates prothrombin in a nonphysiological manner, leading to the formation of fibrin clots [60]. This provides a shield against phagocytes and facilitates abscess formation. Fibrin deposition around bacterial aggregates creates a physical barrier, enabling *S. aureus* to establish a safe haven within the host [60]. Moreover, *S. aureus* can exit the bloodstream and disseminate to different organs. Some toxins, like α-hemolysin, can disrupt endothelial barriers, further facilitating its spread [61]. In certain instances, it may even exploit professional phagocytes as a means of transport, residing within them and using them as “Trojan horses” to reach other tissues (Fig. 2E). Moreover, *S. aureus* has a strong affinity for adhering to foreign materials and forming biofilms [62], which increases the risk of infections in medical devices such as heart valves, prosthetic joints, catheters, and pacemakers.

## Host immune recognition and response to *S. aureus*

**Fig. 3:**
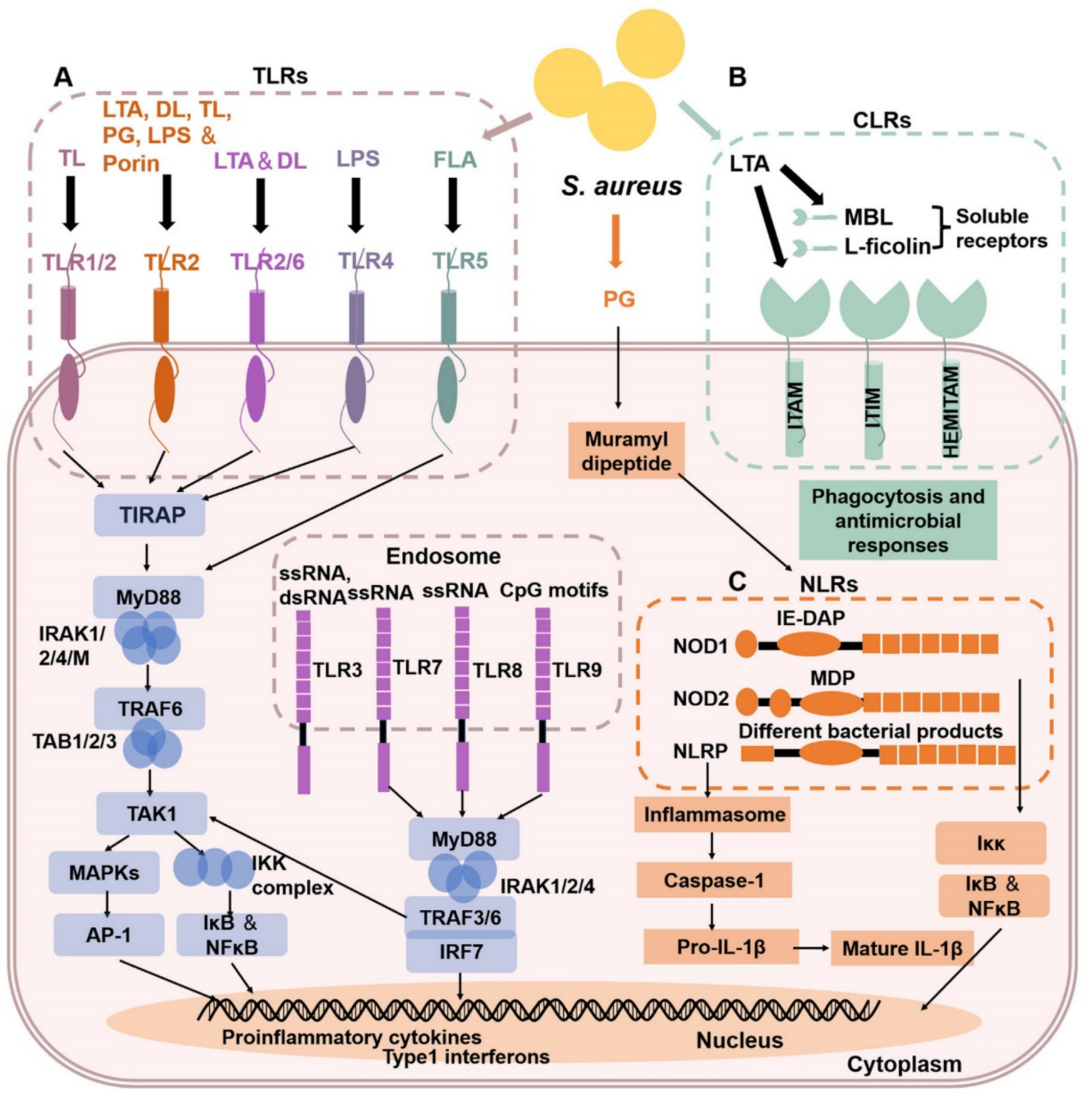
Host recognition of *S. aureus* by the innate immune system. **A. TLRs involved in *S. aureus* recognition.** PAMP-PRR interactions trigger a signaling cascade. TIRAP recruits MyD88, which activates the IRAK complex. IRAK activates TRAF6, which recruits and activates TAK1 with TAB1 − 3. TAK1 then activates IKK-NFκB and MAPK-AP1 pathways. Activated NFκB or AP1 translocates to the nucleus, driving pro-inflammatory cytokine gene transcription. The ssRNA activates TLR7 and TLR8, and CpG-DNA activates TLR9, triggering MyD88-TRAF6-dependent AP1 and NFκB activation, as well as IRAK-, TRAF6-, TRAF3-, and IKKα-dependent IRF7 activation, leading to its translocation and induction of type I interferon genes, including IFNα and IFNβ. **B. CLRs involved in *S. aureus* recognition.** Soluble CLRs, like MBL and L-ficolin, bind pathogen surface proteins. Phosphorylation of cytoplasmic ITIMs recruits phosphatases, while ITAM/hemITAM phosphorylation activates kinase cascades and induces cell activation. Some CLRs lack intracellular motifs, with signaling independent of kinases/phosphatases. CLR activation triggers phagocytosis and antimicrobial responses. **C. NLRs involved in *S. aureus* recognition.** NOD1 and NOD2, located in the cytoplasm, are activated by specific peptidoglycan motifs, with NOD2 recognizing MDP from *S. aureus*. This activation triggers the IKK complex (IKKα, IKKβ, IKKγ/NEMO) to phosphorylate and degrade IκB. This releases NF-κB, which translocates to the nucleus to regulate inflammatory gene expression. NLRPs, especially NLRP3, are activated by *S. aureus* and form the inflammasome, which activates caspase-1 to promote the maturation and secretion of IL-1β.

**Fig. 4:**
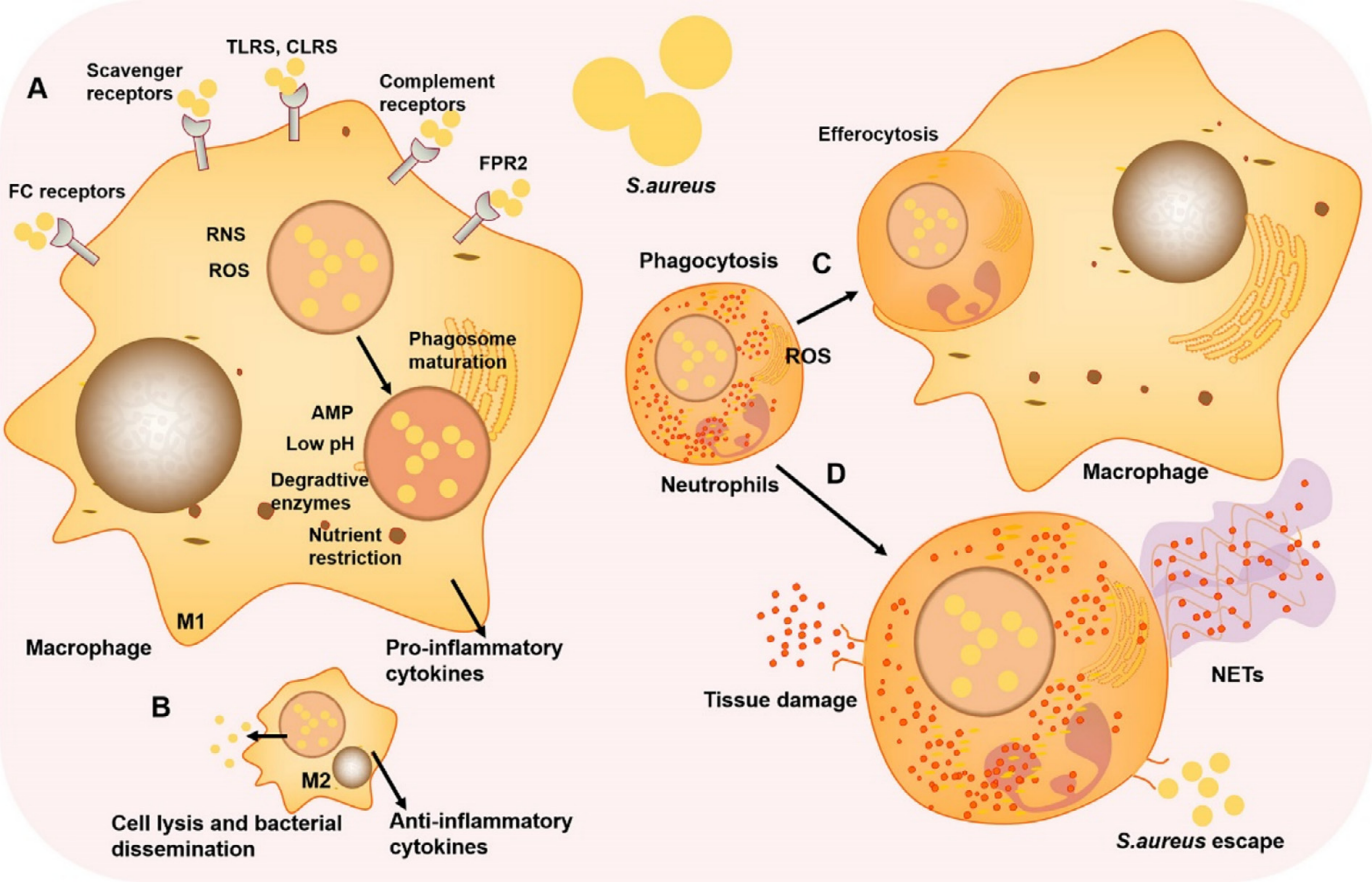
Host immune response to *S. aureus*. **A. The role of M1 macrophages in *S. aureus* infection.** Fc receptors, scavenger receptors, TLRs, CLRs, complement receptors, and FPR2 are several receptors present on the surface of macrophages that recognize *S. aureus*, triggering phagocytosis. Macrophages utilize several mechanisms to degrade *S. aureus* after phagocytosis, including the production of RNS and ROS, the release of degradative enzymes and AMPs, nutrient restriction and phagosome acidification during phagosome maturation. **B. The role of M2 macrophages in *S. aureus* infection.***S. aureus* can evade macrophage defenses by adapting to the phagosome, leading to M2 macrophage lysis and *S. aureus* escape. **C, D. The role of neutrophils in *S. aureus* infection.** Upon migration to the infection site, neutrophils identify and phagocytose invading pathogens. This initiates the generation of ROS and degranulation, both of which contribute to the elimination of *S. aureus*. Following this, neutrophils undergo programmed apoptosis and are subsequently cleared by macrophages (C). Alternatively, *S. aureus* can interfere with neutrophil apoptosis, enhance neutrophil lysis, and facilitate the release of microorganisms. Neutrophils can also generate extracellular DNA networks, known as NETs, at the site of inflammation. These NETs bind pathogens, providing a localized high concentration of antimicrobial molecules that effectively kill the pathogens and form a barrier to prevent their dissemination (D).

*S. aureus* poses a significant threat to the host, and its infection is a complex process involving multiple interactions with the host's innate immune system [28]. The bacterium activates a variety of pattern recognition receptors (PRRs) on immune cells, including TLRs, NOD-like receptors (NLRs), and C-type lectin receptors (CLRs) [63], which recognize PAMPs on its surface (Fig. 3). These interactions trigger a cascade of signaling pathways that stimulate the production of pro-inflammatory cytokines and chemokines, thereby initiating the immune response of host macrophages and neutrophils (Fig. 4). Additionally, *S. aureus* can evade immune detection by secreting various immune-modulatory factors, allowing it to persist within the host and complicate effective immune clearance [63].

TLR2 is a critical receptor for detecting *S. aureus* (Fig. 3A). When the PAMP binds to the extracellular leucine-rich repeats (LRRs) of TLR2, it induces TLR2 heterodimerization, often with TLR1 or TLR6 depending on the nature of the lipopeptide [64]. This dimerization recruits Toll/IL-1 receptor (TIR)-containing adaptor proteins (TIRAPs), which bridge myeloid differentiation primary response gene 88 (MyD88) to TLR2 [65,66]. Subsequently, tumor necrosis factor receptor-associated factor 6 (TRAF6) associates with another complex consisting of transforming growth factor-β-activated kinase-1 (TAK1). This assembly activates two distinct signaling pathways. One pathway is through the IκB kinase (IKK) complex. It is a bottleneck for multiple pathways ultimately leading to NF-κB activation, which is vital for regulating immune and inflammatory responses by inducing the expression of numerous cytokines, chemokines, and antimicrobial effectors [67]. The other pathway involves the TAK1 complex, which activates the mitogen-activated protein kinase (MAPK). These MAPK activations contribute to the induction of activator protein-1 (AP-1), further participating in the regulation of inflammatory and innate immune responses [68]. Similarly, upon stimulation by *S. aureus*, TLR7, TLR8, and TLR9 activate MyD88, recruiting IL-1R-associated kinase (IRAK) and TRAF [69]. TRAF6 activates TAK1, triggering MAPK and IKK pathways (Fig. 3A). TLR9 also activates interferon regulatory factor 7 (IRF7) in plasmacytoid dendritic cells, promoting interferon-alpha (IFNα) transcription [70].

CLRs recognize *S. aureus* by interacting with conserved microbial carbohydrate structures (Fig. 3B). The outcome of CLR recognition of *S. aureus* components depends on their specific signaling motifs. For those with cytoplasmic immunoreceptor tyrosine-based activation motifs (ITAMs/hemITAM), this triggers kinase cascades *via* spleen tyrosine kinase (Syk), leading to cellular activation and the initiation of phagocytosis and antimicrobial responses [63,71]. This activation process helps in the clearance of the pathogen and modulation of the immune response to maintain a balance between pathogen elimination and tissue damage control. While specific evasion factors of *S. aureus* against CLR recognition are largely undescribed, it is known that *S. aureus* can interfere with later stages of complement cascades induced by CLRs [72]. Some CLRs like Clec-60 and Clec-87 have been shown to contribute to host immune responses against staphylococcal infection in certain model systems [63].

NLRs are crucial for sensing *S. aureus*. NOD2, for example, can recognize the peptidoglycan fragment muramyl dipeptide (MDP) from *S. aureus* (Fig. 3C). Upon recognition, NOD2 activates NF-κB signaling pathways [73]. This results in the production of various cytokines and chemokines that are essential for the recruitment and activation of immune cells to combat the *S. aureus* infection. NODs can interact with NOD-, LRR- and pyrin domain-containing proteins (NLRPs), specifically NLRP3. This interaction results in the activation of caspase-1 and the maturation of IL-1β and IL-18 [74].

During *S. aureus* infection, TLRs, NLRs, and CLRs exhibit stage-specific coordination [63]. In acute infection, TLR2/6 synergizes with NLRP3 and NOD2 to drive NF-κB-mediated inflammatory responses (IL-1β/IL-6 production) and neutrophil recruitment, while CLRs including mannose-binding lectin (MBL) enhance bacterial clearance through opsonization, complement activation, and promotion of macrophage phagocytosis [63,75]. In chronic infection, *S. aureus* evades immune responses *via* virulence factors and biofilms, which suppress TLR2 signaling. Prolonged NLRP3 activation leads to dysregulated IL-1β release, contributing to tissue damage, while NOD2-mediated autophagy exacerbates inflammation [76]. In summary, immune regulation holds promise as a key strategy in HDTs to suppress *S. aureus*.

## HDTs to suppress *S. aureus* infections

### Improving host immunity to suppress *S. aureus* infections

The immune system plays a critical role in defending the body against infections. However, under prolonged evolutionary pressure, many bacteria, including *S. aureus*, have developed sophisticated mechanisms that produce a significant variety of virulence and immune evasion factors (such as phenol-soluble modulins, staphylococcal superantigens, staphylococcal peroxidase inhibitors, etc.) to circumvent host immune defenses and antibiotic therapy [28,77]. This immune evasion significantly complicates the treatment of *S. aureus* infections, particularly those involving intracellular bacteria [78,79]. Targeting immune cells such as macrophages and neutrophils, or cellular organelles like lysosomes, to restore or enhance their function holds great potential for activating the host immune response and improving the control of *S. aureus* infections [31,80].

#### Polypeptides

Host defense peptides (HDPs) are a class of nutritional or immunomodulatory molecules that harness natural host mechanisms to enhance therapeutic benefits and have garnered widespread attention [34,81]. Certain HDPs can activate immune cells such as macrophages and neutrophils [82], thereby enhancing host's bacterial killing capacity. For example, a specific formyl peptide receptor 2 agonist TRP-LYS-TYR-MET-VAL-D-MET (WKYMVm) can stimulate human monocytes to produce superoxide and kill *S. aureus*, while also acting as a chemotactic factor to recruit phagocytes [83]. Additionally, LL-37 promotes dendritic cell maturation and enhances the phagocytic activity of macrophages and neutrophils. It boosts neutrophil extracellular trap (NET) formation, effectively trapping *S. aureus* [84].

As interest in peptides grows, some have been isolated from organisms for their immune-modulating and anti-*S. aureus* activities, with several also exhibiting broad-spectrum antimicrobial properties. Pituitary adenylate cyclase activating polypeptide is induced in the brain in response to bacterial and fungal infections and shares almost identical structural similarities with the human LL-37. It interacts with the immune system to modulate immune responses and has been shown to exert anti-inflammatory and neuroprotective effects in mouse models of multiple sclerosis [85]. Furthermore, Gy-CATH is a newly discovered antimicrobial peptide isolated from the skin of *Glyphoglossus yunnanensis*. While it does not have direct antibacterial properties, it has shown significant preventive and therapeutic effects in mice infected with *S. aureus*, MRSA, *Enterobacteriaceae coli* and carbapenem-resistant *Escherichia coli* (CREC) [86]. Similar to Gy-CATH, which modulates immune responses, Nv-CATH also plays a crucial role in immune defense. It is a novel 30-residue peptide from the frog *Nanorana ventripunctata*, exhibits broad-spectrum antimicrobial activity against both Gram-positive and Gram-negative bacteria. It enhances immune defense by promoting neutrophil phagocytosis, NET formation, and recruiting immune cells to the infection site. Its dual antimicrobial and immunomodulatory mechanisms make it a promising candidate for the development of new antimicrobial agents to combat antibiotic resistance [87].

In the pursuit of alternative treatments to antibiotics, targeting tumor necrosis factor receptors (TNFRs) have emerged as a promising therapeutic strategy for combating bacterial skin infections. Mechanistically, TNFR1 promotes the formation of neutrophilic abscesses in the skin, which is essential for host defense against *S. aureus*. On the other hand, TNFR2 signaling is associated with the formation of NETs, which are structures that can trap and kill microbes, and the generation of ROS *via* nicotinamide adenine dinucleotide phosphate (NADPH) oxidase 2 activation, further amplifying the antimicrobial response [88]. It is important to note that patients undergoing TNF inhibitor therapy exhibit a higher susceptibility to *S. aureus* skin colonization and subsequent infection. While the treatment with the TNFR2 agonist resulted in reduced skin lesion sizes and bacterial burdens [88,89]. For example, a single intraperitoneal dose (8 mg/kg) of the TNFR2 agonist NewSTAR2 significantly reduced skin lesion size and bacterial burden in *S. aureus*-infected mice compared to vehicle controls. Notably, this compound exhibited no direct antibacterial activity against *S. aureus*. It enhanced NET formation in infected skin tissue, which may correlate with improved bacterial clearance [88].

#### Small molecules

In addition to peptides, small molecules can also regulate the host's immune response against *S. aureus*. Small molecule inhibitors targeting deoxycytidine kinase (dCK), such as (R)-DI-87, can help prevent immune cell death during *S. aureus* infections. *S. aureus* secretes nuclease that degrades NETs, releasing toxic deoxyribonucleosides like deoxyadenosine and deoxyguanosine. These are converted into deoxyadenosine triphosphate and deoxyguanosine triphosphate inside phagocytes, triggering apoptosis. By inhibiting dCK, these inhibitors block the conversion of deoxyadenosine and deoxyguanosine into toxic nucleotides, thus preserving macrophage integrity and mitochondrial function. This sustains macrophage migration to infection sites while enhancing bactericidal activity (ROS production, lysosomal fusion), improving bacterial clearance with reduced host tissue damage [90]. Moreover, N-phosphonacetyl-L-aspartate (PALA) enhances bacterial clearance in normal human dermal fibroblasts by activating the NOD2 signaling pathway, highlighting its potential for treating skin infections. While PALA does not have direct bactericidal effects on MRSA, it boosts host immune responses without being cytotoxic to normal human dermal fibroblasts [91].

*S. aureus* evades autophagic degradation by impairing lysosomal function and disrupting autophagic flux during cellular invasion [92]. Thus, targeting lysosomal activation to restore its function represents a promising approach in HDTs. The molecule TTTh specifically targets the lysosomes and promotes their maturation to accelerate the clearance of MRSA. It eradicates the intracellular bacteria by triggering ROS accumulation and compromising membrane integrity. Wounds treated with TTTh show a reduction in bacterial load and improved healing [32]. Additionally, several agents that do not exhibit direct antibacterial properties, such as OG9 [93], gamma-aminobutyric acid (GABA) [94], 2-deoxyglucose [95], and vitamin D [96], have been shown to eradicate the intracellular bacteria by enhancing lysosome biogenesis and acidification. As a result, the identification and development of lysosome-directed agents offer promising candidates for HTDs to combat bacterial infections.

Additionally, various host-directed repurposing strategies of existing drugs have shown efficacy in alleviating bacterial infections. The irreversible pan-caspase inhibitor emricasan has capacity to modulate host-pathogen interactions, immune responses, and cellular metabolic processes, enhancing its potential as a therapeutic strategy for *S. aureus* infections [97]. Another pan-caspase inhibitor, Q-VD-OPH has demonstrated therapeutic efficacy against *S. aureus* skin infections in mice by reducing apoptosis in monocytes and neutrophils through the inhibition of caspases-3, −8, and −9 [98]. Lbrutinib, a tyrosine kinase inhibitor, enhanced host cell viability without affecting bacterial growth and reduced intracellular bacterial recovery, particularly at early time points. It inhibited phagosomal escape, decreasing bacterial presence in the cytosol. Phosphoproteomic analysis identified key pathways modulated by Ibrutinib, including focal adhesion and MAPK signaling. Ibrutinib's effect was linked to the phosphorylation of c-JUN and EPHA2, with EPHA2 knockout restoring host cell viability and reducing bacterial load. Other tyrosine kinase inhibitors, dasatinib and crizotinib, also targeted host pathways to reduce *S. aureus* burden, with dasatinib disrupting multiple kinases and receptors to impair bacterial survival. Crizotinib also interferes with host factors crucial for bacterial replication, although its exact mechanism is less clear. Further studies are needed to clarify the molecular mechanisms of these inhibitors [33]. Ticagrelor, an antiplatelet agent, inhibits *S. aureus* through direct antibacterial activity. It targets the platelet adenosine diphosphate P2Y12 receptor, enhancing platelet-mediated killing of *S. aureus*, preventing α-toxin-induced desialylation to alleviate thrombocytopenia, and inhibiting bacterial adhesion to endothelial tissues by preventing platelet aggregation [99]. It is rapidly absorbed orally, with peak plasma concentrations reached at 1.3 to 2 h. It is predominantly metabolized by CYP3A4 (95 % contribution) to form an active metabolite, with minor involvement of CYP3A5 [100].

#### Metallic species

Some metallic species also regulate bacterial infections, which are of significant importance in drug development [101]. For example, levels of ferrous iron and ferroptosis-related biomarkers fluctuate in cells during bacterial infection [102]. Experiments have shown that ferrous iron is transported into the bacterial compartment within macrophages *via* ferroportin, where it triggers a ferroptosis-like death in the bacteria. Therefore, the use of ferroptosis inducers, such as acetaminophen, sulfasalazine, or ras-selective lethal small molecule 3, holds potential for enhancing iron toxicity and inhibiting bacterial infection *in vivo* [103]. In addition, transferrin is essential for nutritional immunity by competitively binding iron, limiting bacterial access to this crucial nutrient [104]. Studies show that transferrin improves survival rates and reduces bacterial burden in infected mice, and when used in combination with other drugs, it effectively restricts the development of resistance [105]. Therefore, the development of transferrin-activating agents holds promise as a novel antimicrobial strategy that warrants further clinical investigation.

Similarly, copper ions also exhibit antimicrobial properties that can be utilized by macrophages to enhance immune responses. Therefore, copper-activated compounds, such as glyoxal-bis(N4-methylthiosemicarbazone), have the potential to facilitate the elimination of MRSA [106]. Moreover, silver, zinc, gallium, manganese, and magnesium have long been recognized for their ability to modulate bacterial infections [107,108]. Today, metal particles remain a key focus in the development of bioactive materials, owing to their ability to generate stable, cost-effective, and broad-spectrum antibacterial surfaces for implants [35].

#### Nanoparticles

Furthermore, the strategy of targeting the host to deliver drugs that combat bacterial infections has proven to be highly effective and promising, particularly through the reprogramming of immune cells such as macrophages. In the case of sepsis caused by MRSA, a major challenge is the pathogen's ability to evade the immune system. To address this, Tang et al. developed CRV peptide-modified lipid nanoparticles (CRV/LNP-RNAs) that deliver chimeric antigen receptor mRNA targeting MRSA, along with caspases-11 siRNA, which disrupts MRSA's intracellular immune evasion mechanisms [109]. An adamantane (Ada)-based antibiotic-peptide conjugate, Cip-CBT-Ada, is designed for targeted intracellular delivery. Ciprofloxacin is coupled with a β-cyclodextrin-heptamannoside (CD-M) derivative, forming a complex (Cip-CBT-Ada/CD-M) that leverages multivalent ligand-receptor interactions to target macrophages overexpressing the mannose receptor [110].

An increasing number of phytochemicals are being incorporated into nanotechnology-based systems to better target immune cells and enhance the activation of host immunity. Isoliquiritigenin-loaded nanoparticles modulate host immunity to combat *S. aureus* through multiple mechanisms. Isoliquiritigenin alleviates inflammation by suppressing pro-inflammatory cytokines (e.g., TNF-α, IL-6), thereby reducing host tissue damage. In a murine model of MRSA biofilm implant infection, the treatment effectively reduced bacterial burden and mitigated inflammation without significant toxicity [111,112]. Hyaluronic acid-streptomycin-lipoic acid-glabrol nanoparticles targets host immune cells infected with Staphylococcus aureus by binding to CD44 receptor on macrophages, delivering the glabrol-streptomycin combination *via* glutathione-responsive release. It reduces intracellular bacterial burden by 2 to 10-fold in murine models while promoting wound healing with low toxicity [113]. The glabridin-cinnamaldehyde-dextran nanoparticles target macrophages due to dextran's affinity for macrophage lectins. Inside macrophages, the acidic lysosomal environment cleaves the pH-sensitive acetal linkages, releasing cinnamaldehyde and glabridin. Cinnamaldehyde inhibits MRSA toxin expression, reducing macrophage damage and bacterial spread, while glabridin effectively kills intracellular MRSA, preventing bacterial growth and dissemination [114].

### Regulating host oxidative stress to suppress *S. aureus* infections

ROS refer to a group of highly reactive molecules, including hydrogen peroxide (H_2_O_2_), molecular oxygen (O_2_), hydroxyl radicals (•OH), and superoxide anion (O_2_•−). These molecules exert antimicrobial effects against both Gram-positive and Gram-negative bacteria, including multidrug-resistant strains. This action occurs through the induction of oxidative stress, which results from an imbalance between ROS levels and the antioxidant defense mechanisms that neutralize them. The oxidative stress driven by ROS can lead to damage of critical cellular components such as DNA, lipids, and proteins. Their highly reactive nature makes them key players in the immune defense against *S. aureus*. During an infection, neutrophils quickly engulf *S. aureus* and release ROS as part of the respiratory burst. This mechanism is particularly crucial, as demonstrated by the high incidence of *S. aureus* infections in individuals with chronic granulomatous disease, a genetic disorder caused by a defective phagocyte respiratory burst oxidase, which impairs ROS production due to a faulty NADPH oxidase enzyme [115]. Additionally, studies show that mice lacking NADPH oxidase exhibit greater susceptibility to *S. aureus* infections [116], while the use of the NADPH oxidase inhibitor diphenyleneiodonium in isolated human neutrophils significantly reduces their ability to eliminate *S. aureus* [115]. This highlights the vital role of ROS and NADPH oxidase enzyme in the host's defense against this pathogen. Thus, increasing ROS production may potentially lead to new treatment options and enhance antibacterial therapies. However, it is important to exercise caution, as low levels of ROS can actually benefit bacteria and contribute to the development of resistance [117].

In addition, HDTs that harness ROS can enhance drug targeting accuracy while minimizing toxic effects. Studies indicate that pathogen invasion induces changes in the redox potential of host cells, which can serve as a distinguishing feature between infected and uninfected cells. In the case of *S. aureus*-infected osteoblasts, increased expression of NAD-related genes and enhanced surface reduction activity indicate a shift towards higher oxidative-reductive metabolic activity in the infected cells. These redox changes, particularly in NADH/NAD^+^, NADPH/NADP^+^, and reduced glutathione/oxidized glutathione ratios, are significantly elevated in infected cells compared to uninfected ones. These findings suggest that the altered redox state could be harnessed for developing reduction-responsive drugs, providing a novel approach to selectively target and treat intracellular bacterial infections [118].

### Alleviating host inflammation to suppress *S. aureus* infections

*S. aureus* triggers various inflammation-related diseases, causing damage to the host. Therefore, targeting the host to alleviate inflammation is crucial in clinical treatment strategies. The novel molecular entity targeting the NLRP3 inflammasome, 1-peptidyl-2-arachidonoyl-3-stearoyl-*sn*-glyderide, has been shown to reduce inflammation and pathogen burden in infections such as MRSA and Gram-negative bacteremia. It demonstrates superior efficacy compared to standard antibiotics in treating multifactorial bacterial infections [119]. The virulence factor α-toxin of *S. aureus* activates NLRP3 in macrophages, inducing a glycolytic shift that depletes glucose in host cells and promotes bacterial survival under antibiotic pressure. Antibiotic tolerance may develop when *S. aureus* transition into a reduced metabolic state marked by decreased ATP levels [[120], [121], [122]]. By inhibiting NLRP3 with MCC950, this glycolytic shift is diminished, leading to improved glucose availability for the bacteria, which sensitizes *S. aureus* to antibiotics. In murine models, MCC950 pretreatment enhances the efficacy of rifampicin treatment, particularly in infections involving α-toxin-producing *S. aureus* strains. These findings suggest that targeting NLRP3 can be a promising strategy to combat antibiotic tolerance in bacterial infections [123]. Moreover, OLT1177, an approved inflammasome inhibitor with established safety in human clinical trials, functions by blocking the activation of NLRP3. It likely blocks the signals or events that trigger NLRP3 activation, such as the interaction between danger-associated molecular patterns or PAMPs and NLRP3. By inhibiting NLRP3 activation, OLT1177 reduces the production and release of IL-1β, thereby mitigating the inflammatory response within the joint. This dual effect of reducing inflammation and protecting cartilage makes OLT1177 a promising adjuvant therapy in combination with antibiotics like rifampin for treating septic arthritis caused by MRSA [124].

In addition to NLRP3, several host proteins are also crucial in mediating the inflammatory response triggered by *S. aureus* infection. Phosphodiesterase 4 (PDE4) is an enzyme primarily expressed in keratinocytes and immune cells, where it hydrolyzes cyclic adenosine 3′, 5′-monophosphate and promotes pro-inflammatory pathways upon activation. PDE4 inhibitors have been developed and are now approved as host-directed antimicrobial drugs (HDADs) for several inflammatory conditions [125], including chronic obstructive pulmonary disease [126], psoriasis [127], and AD [128]. Among them, crisaborole has been approved for the topical treatment of mild-to-moderate AD, with clinical improvements linked to lower levels of inflammatory markers, reduced epidermal thickness, and enhanced skin barrier function [129,130]. Furthermore, a 2 % crisaborole ointment is well-tolerated and offers benefits in patients with facial and anogenital psoriasis [131,132].

In a mouse model of AD-like skin inflammation induced by MRSA, treatment with crisaborole reduced skin colony-forming units both when used alone and in combination with oral linezolid, with the combination showing enhanced efficacy. Mechanistically, crisaborole inhibits PDE4-mediated cyclic adenosine monophosphate degradation, thereby elevating its levels in immune cells (macrophages/neutrophils). This suppresses pro-inflammatory pathways (NF-κB, Th1/Th17) and myeloid cell recruitment, while reducing IL-1α-driven keratinocyte hyperproliferation and enhancing barrier function. Collectively, these host-directed actions remodel the skin microenvironment to limit *S. aureus* colonization despite lacking direct bactericidal activity [125].

TLRs and their downstream pathways are key players in the host's regulation of the inflammatory response to *S. aureus* infections. Keratin 6a-derived antimicrobial peptides (KAMPs) were shown to interfere with bacterial signaling by competing with bacterial ligands for cell surface TLRs and co-receptors, including MD-2, CD14, and TLR2. Additionally, KAMPs promoted the endocytosis of these receptors, thereby reducing their surface availability and modulating the host's inflammatory response. Importantly, the topical application of KAMPs in an experimental model of bacterial keratitis resulted in significant therapeutic benefits, including reduced corneal opacity, diminished inflammatory cell infiltration, and decreased bacterial load [133]. Individuals with diabetes mellitus are at an increased risk of infection by *S. aureus*, particularly in cases of skin infections and pneumonia. Managing these infections is challenging due to limited treatment options [134]. The combination of linezolid and insulin demonstrated significant antibacterial and anti-inflammatory effects, targeting the TLR2/MAPKs/NLRP3 pathway. These findings offer promising therapeutic options for *S. aureus* infections in diabetic patients [135].

Baicalin (BAI), a flavonoid from Radix Scutellariae (Huang Qin), shows potential as a HDT against MRSA-induced sepsis. Sepsis, caused by infections like MRSA, involves a dysregulated host immune response. BAI has anti-inflammatory, antioxidant, and antitumor properties [136]. In MRSA infection, BAI inhibits pro-inflammatory cytokines (e.g., IL-6, TNF-α) from macrophages and dendritic cells by blocking the ERK, JNK, MAPK, and NF-κB pathways. In mice, BAI reduced TNF-α and increased IL-10, promoting immune regulation. When combined with vancomycin, BAI further lowered bacterial load in organs and reduced tissue damage [137]. BAI shows moderate oral bioavailability (40.12 %) and favorable drug-likeness (score 0.77). It is likely metabolized in the liver and renally excreted, supporting its potential for fatty liver treatment [138]. However, detailed metabolic pathways and excretion kinetics need further investigation. These results suggest that BAI could be a promising HDT strategy for combating MRSA and other antibiotic-resistant infections. In addition, brazilin treatment in a mouse model and *S. aureus* mastitis cells suppressed the overexpression of TLR2 by regulating the NF-κB and MAPK signaling pathways, which in turn attenuated inflammatory cell infiltration and inhibited cytokine expression, making it an effective treatment for mastitis [139].

Hydroxycarboxylic acid receptor 2 (HCAR2) has emerged as a prominent target for regulating inflammation in recent years and has been found to play a role in mitigating *S. aureus* infections. Upon activation, HCAR2 inhibits the expression of cytidine/uridine monophosphate kinase 2, which in turn reduces mitochondrial DNA (mtDNA) replication, alleviates mitochondrial damage, and suppresses the activation of the NLRP3 inflammasome and subsequent pyroptosis [140]. As a result, the integrity of the blood-milk barrier is preserved. Furthermore, the decreased release of mtDNA from mammary epithelial cells prevents the activation of the cyclic GMP-AMP synthase/stimulator of interferon gene pathway in macrophages, thereby maintaining macrophage function and bactericidal activity, which effectively reduces the inflammatory response [141]. HCAR2 agonists, such as nicotinic acid, have been shown to alleviate the pathological changes in mammary gland tissue induced by *S. aureus* infection [140].

### Repairing skin barrier to suppress *S. aureus* infections

Human beta-defensin 2 (HBD2) plays a crucial role in protecting the skin barrier from damage caused by *S. aureus*. Secretomic analysis revealed a significant upregulation of Laminin subunit beta-1 in HBD2-treated samples, compared to those exposed to V8 alone. Similarly, proteomic analysis identified elevated levels of Carcinoembryonic antigen-related cell adhesion molecule 6 and LEPREL1 (a protein involved in collagen biosynthesis), in cells treated with V8 and protected by HBD2. These changes in protein expression suggest that HBD2 may modulate the host’s protein synthesis and secretion pathways, particularly those related to the extracellular matrix (ECM). Such modulation could enhance the capacity of keratinocytes to maintain ECM integrity, thereby protecting the skin barrier from V8 protease-mediated damage [142]. Furthermore, certain agents can protect the skin barrier by promoting the secretion of HBD from the host. In rat models, the protein-rich fraction from *Lactobacillus plantarum* USM8613 also exhibited wound-healing properties. Notably, USM8613 significantly upregulated HBD expression by 3.8-fold and enhanced cytokine and chemokine production during wound healing. Further experiments using Δ*atl S. aureus* showed that its inhibitory effect was mediated by targeting the *atl* gene [143]. Rhein-loaded chitosan nanoparticles were designed to provide broad-spectrum anti-infection effects and facilitate the healing of MRSA-infected wounds by promoting fibroblast migration and angiogenesis, both of which are critical for tissue repair and regeneration [144].

Some HDADs are crucial for maintaining skin immune function and restricting *S. aureus* colonization. Research has indicated that a deficiency in Vitamin A can compromise immune responses, leading to a heightened vulnerability to skin infections and inflammatory conditions [145]. Moreover, Vitamin A enhances the expression of RELMα in keratinocytes, a protein with antimicrobial properties *in vitro*, which also helps protect against bacterial infections in the skin *in vivo* [145]. Vitamin A plays a role in upregulating TLR expression, and influences the proliferation of keratinocytes and the activity of Langerhans cells and mast cells [146]. Furthermore, the PDE4 inhibitor crisaborole may help preserve the skin barrier, potentially limiting *S. aureus* colonization, and could serve as a promising new topical therapy in the development of treatments for AD [125]. The synthetic peptide DRGN-1, based on a natural host defense molecule of the Komodo dragon, accelerates healing of biofilm-infected cutaneous wounds through enhanced keratinocyte migration and activation of the epidermal growth factor receptor (EGFR)-signal transducer and activator of transcription (STAT) 1/3 signaling pathway [147].

### Enhancing cell membrane permeability to suppress *S. aureus* infections

A novel therapeutic strategy has been proposed for effectively combating intracellular *S. aureus* infections using NK cell mimics (NKMs), which employ a dual-target mechanism to enhance antibacterial efficacy. NKMs are engineered by loading perforin and granzyme B into mesoporous silica nanoparticles, which are activated by the redox changes in the infected cell environment. Upon activation, perforin creates pores in the infected cell membrane, allowing granzyme B to enter and kill the pathogens. This mechanism mimics the action of NK cells in clearing intracellular infections. NKMs demonstrate significant efficacy against vancomycin-insensitive *S. aureus*, enhance immune responses, and promote long-lasting protective immunity. This approach offers advantages such as easy production, high biosafety, and sustained therapeutic effects, making it a promising strategy for treating intracellular bacterial infections [118]. Similarly, alnustone treatment enhances the sensitivity of MRSA to antibiotics and boosts the host immune response, which is associated with an increase in cell membrane permeability. In a murine model of MRSA infection, the combination of alnustone and antibiotics notably improved treatment efficacy, leading to a significant reduction in bacterial burden and alleviation of tissue damage [148].

### Regulating cellular autophagy to suppress *S. aureus* infections

Intracellular *S. aureus* infection induces metabolic alterations in host cells, triggering autophagy that the bacteria exploit for survival [149]. Adenosine monophosphate-activated protein kinase (AMPK) and extracellular signal-regulated kinase (ERK) are key sensors in this process [150]. AMPK activation during *S. aureus* infection triggers autophagy (*via* ULK1 phosphorylation) to replenish nutrients, inadvertently sustaining bacterial replication. Conversely, AMPK inhibition disrupts this metabolic hijacking, reducing intracellular bacterial survival. Strikingly, both strategies show therapeutic potential—activation enhances neutrophil chemotaxis and bacterial killing in later stages of *S. aureus* infection [151], while early inhibition blocks pathogen exploitation of host resources—revealing context-dependent opportunities for HDTs [152].

Inhibition of the host AMPK pathway by dorsomorphin significantly impairs intracellular MRSA survival. Mechanistically, dorsomorphin blocks AMPK phosphorylation, reducing the conversion of LC3-I to LC3-II (autophagy markers) and impairing autophagic flux. This disruption of the starvation-induced autophagy pathway starves intracellular bacteria of nutrients derived from host macromolecule degradation. Experimental evidence in MRSA-infected HeLa cells and human umbilical vein endothelial cells demonstrates that dorsomorphin treatment reduces autophagy levels while increasing host cell viability and decreasing bacterial survival. Notably, the antimicrobial effect is more pronounced in human umbilical vein endothelial cells, suggesting cell-type specific responses. These findings indicate that AMPK inhibition disrupts MRSA's ability to utilize autophagy-induced nutrient scavenging and energy generation, thereby limiting its intracellular proliferation. This represents a promising HDT that operates without direct bacterial targeting [152].

It is revealed that some ATP-competitive kinase inhibitors can reduce intracellular MRSA by activating AMPK-mediated autophagy. The researchers screened ATP-competitive kinase inhibitors from the Published Kinase Inhibitor Sets (PKIS1 and PKIS2), identifying 17 compounds that reduced bacterial burden. Among these, GW296115X, a staurosporine derivative, was particularly effective at decreasing intracellular MRSA load, with an IC_50_ of 159 nM. This effect was mediated by the activation of AMPK, which promoted autophagy and enhanced bacterial degradation. GW296115X primarily targets AMPK, and AMPK-related kinases such as BRSK1, BRSK2, and Nuak1. Notably, the antibacterial effect of GW296115X was completely reversed when autophagic flux was inhibited by bafilomycin A1, underscoring the critical role of autophagy in its HDT mechanism. In addition to GW296115X, GW633459A also demonstrated host-directed activity against intracellular MRSA, though its mechanism of action was independent of its known targets in the EGFR/HER kinase family. The precise targets of GW633459A remain unclear and require further investigation. Both compounds exhibited low toxicity in zebrafish embryo models, with GW296115X significantly improving survival rates in MRSA-infected embryos, while GW633459A had a more moderate effect [153].

Statins, primarily known for lowering cholesterol, have gained attention for their potential role in combating infections, including *S. aureus*. These drugs work by inhibiting 3-hydroxy-3-methylglutaryl coenzyme A (HMG-CoA) reductase, a key enzyme in the cholesterol synthesis pathway [154]. This inhibition not only decreases cholesterol levels but also reduces the production of intermediates like farnesyl pyrophosphate and geranylgeranyl pyrophosphate. These intermediates are critical for the post-translational modification (prenylation) of small GTPases, which are involved in various cellular processes such as membrane anchoring and signaling [155,156]. Pathogenic bacteria, including *S. aureus*, utilize these modifications to facilitate host cell invasion and establish infections. By blocking the formation of these intermediates, statins hinder the bacteria's ability to interact with host cells and disrupt their invasive processes. Additionally, statins may enhance the host immune response, offering a dual mechanism of action in the treatment of infections. Thus, the effects of statins extend beyond cholesterol lowering, potentially offering a valuable strategy in infectious disease management [156].

Translocation-associated membrane protein 2 (TRAM2) depletion or thapsigargin treatment reduced autophagy in infected cells. The researchers identified TRAM2, a gene involved in polypeptide transport across the endoplasmic reticulum membrane, as a key factor in *S. aureus* infection. TRAM2 interacts with the ER Ca^2+^ pump sarcoplasmic/endoplasmic reticulum calcium ATPase (SERCA) 2b, which is essential for collagen type I folding, and its protein levels were found to change during infection, suggesting that *S. aureus* may alter its post-translational modification. The study also found that low concentrations of thapsigargin, a SERCA inhibitor, enhanced host cell viability and reduced intracellular MRSA survival, without directly affecting bacterial growth *in vitro*. Although the exact mechanism remains unclear, these findings highlight the potential of thapsigargin as a HDT, particularly when combined with conventional antibiotics, by targeting host factors such as TRAM2 and the SERCA pump to disrupt the intracellular survival of *S. aureus* [157].

## Key host targets and their active pockets for drug design

Drug target discovery is crucial for developing new drugs [158]. By identifying the specific molecules in biological tissues and body fluids that interact with drugs, researchers can design more effective HDADs [159,160]. Important aspects of drug targets are binding sites within their active pockets and binding energy. Binding sites play a vital role as they are directly involved in binding with the drug molecules. Understanding and characterizing these amino acid sites can provide insights into the mechanism of drug action and help in the design of more potent and selective drugs. Binding energy provides information on the ease of ligand–protein binding and the strength of the interaction. Computer-aided drug design (CADD) has been widely applied in recent years. It offers several advantages in the discovery of novel targeted-therapy drugs. CADD can predict the binding affinity and orientation of drugs to their targets, allowing for the screening of large libraries of compounds to identify potential leads [161]. It can also provide detailed structural information about the drug-target interaction, facilitating the optimization of drug candidates. Additionally, CADD can save time and resources by reducing the need for extensive experimental screening [162].

**Fig. 5:**
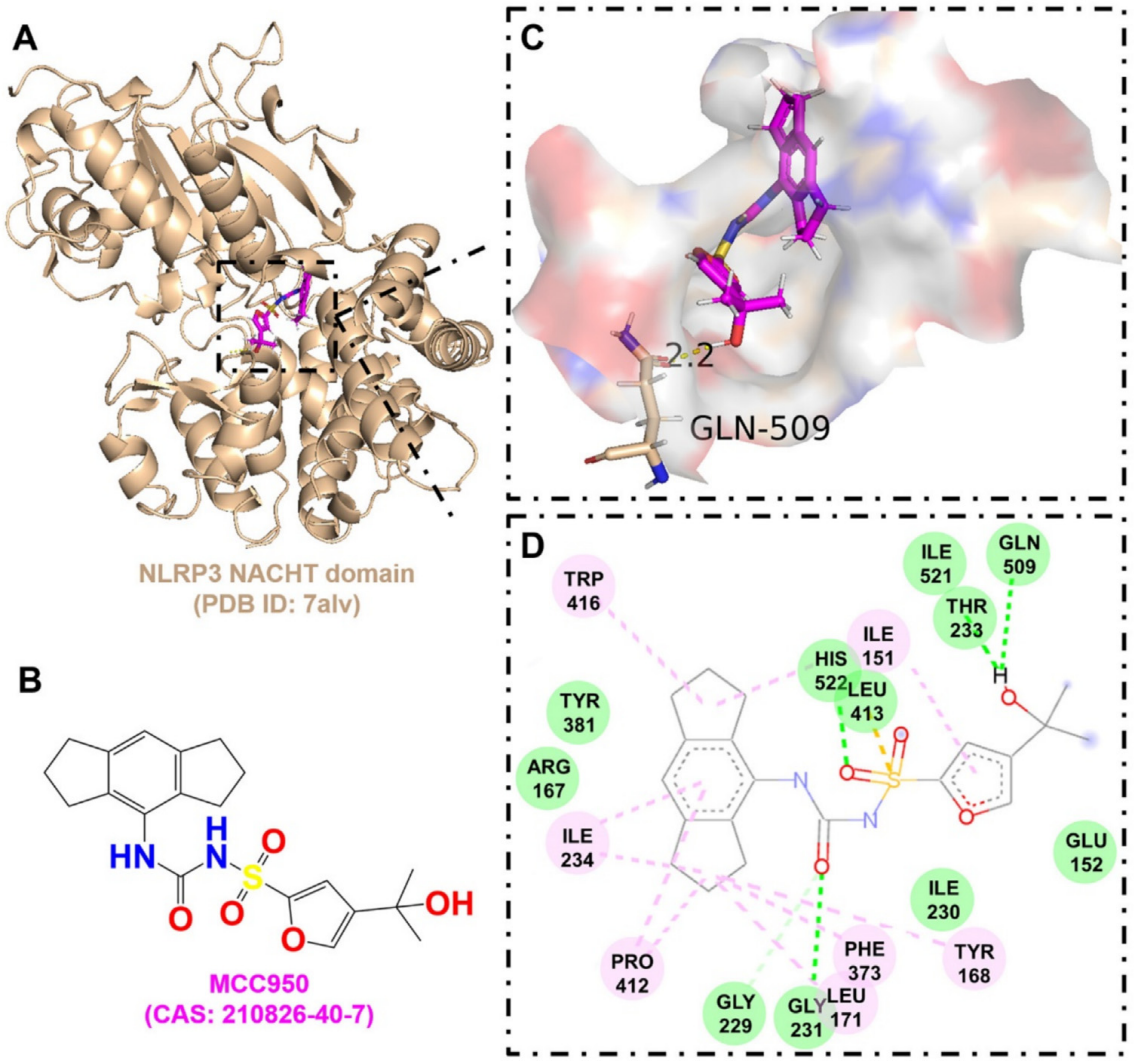
The interaction between MCC950 and the NLRP3 NACHT domain. A. The 3D cartoon model of overall structure of the NLRP3 NACHT domain (PDB ID: 7alv). B. The chemical formula of MCC950 (CAS: 210826–40-7). C. The close-up view of the interaction between MCC950 and the NLRP3 NACHT domain. The residues involved in the binding are shown in wheat (stick model) with their names labelled, while the ligand is shown in magenta. The hydrogen bonds are drawn as yellow dashed lines, with distances labeled. The images were generated using PyMOL software. D. The 2D interaction postures were also generated with van der Waals interactions, carbon hydrogen bond, pi-alkyl, and pi-sulfur shown in green, light green, pink, and yellow dashed lines, respectively. NLRP, NOD-, LRR- and pyrin domain-containing protein. (For interpretation of the references to colour in this figure legend, the reader is referred to the web version of this article.)

The therapeutic targeting of NLRP3 has been hindered by an insufficient understanding of its structure and mechanism of action [163]. Nevertheless, recent research has provided valuable new insights into the structural characteristics, activation processes, and signaling pathways of NLRP3, paving the way for potential therapeutic strategies [[164], [165], [166], [167], [168], [169], [170]]. A 2.8 Å resolution crystal structure was obtained for the NACHT domain of NLRP3, a central ATPase domain, in complex with an MCC950 analog [165]. Further biophysical and biochemical analyses demonstrated that MCC950 and its analogs interact with the nucleotide-binding motifs in the NACHT domain, likely disrupting ATP/ADP binding and ATP hydrolysis [166,171]. However, there is currently insufficient research on the active binding pocket of NLRP3 in complex with MCC950. Molecular docking simulations suggest that MCC950 may interact with the NACHT domain, specifically through hydrogen bonding with GLN-509 (Fig. 5), with a binding energy of −8.60 kJ/mol (Table 2). This indicates that targeting this region could offer potential for the development of small molecule inhibitors aimed at alleviating *S. aureus* infections through NLRP3 modulation.

**Table 2:** Drug-target interaction information.

Target	Agent	Key binding residues	Score	Binding energy (kJ/mol)
AMPK	Dorsomorphin	VAL-130	6.94	−8.78
Deoxycytidine kinase	(R)-DI-87	SER-35, GLN-97, ARG-128, ASP-133	7.12	−8.88
GAPDH	4-Octyl itaconate	THR-211	6.12	−7.38
NLRP3	MCC950	GLN-509	7.81	−8.60
Phosphodiesterase 4	Crisaborole	ASP-564, ASN-567, GLN-615	6.46	−6.12
TLR2	Brazilin	TYR-715	5.12	−6.22

**Fig. 6:**
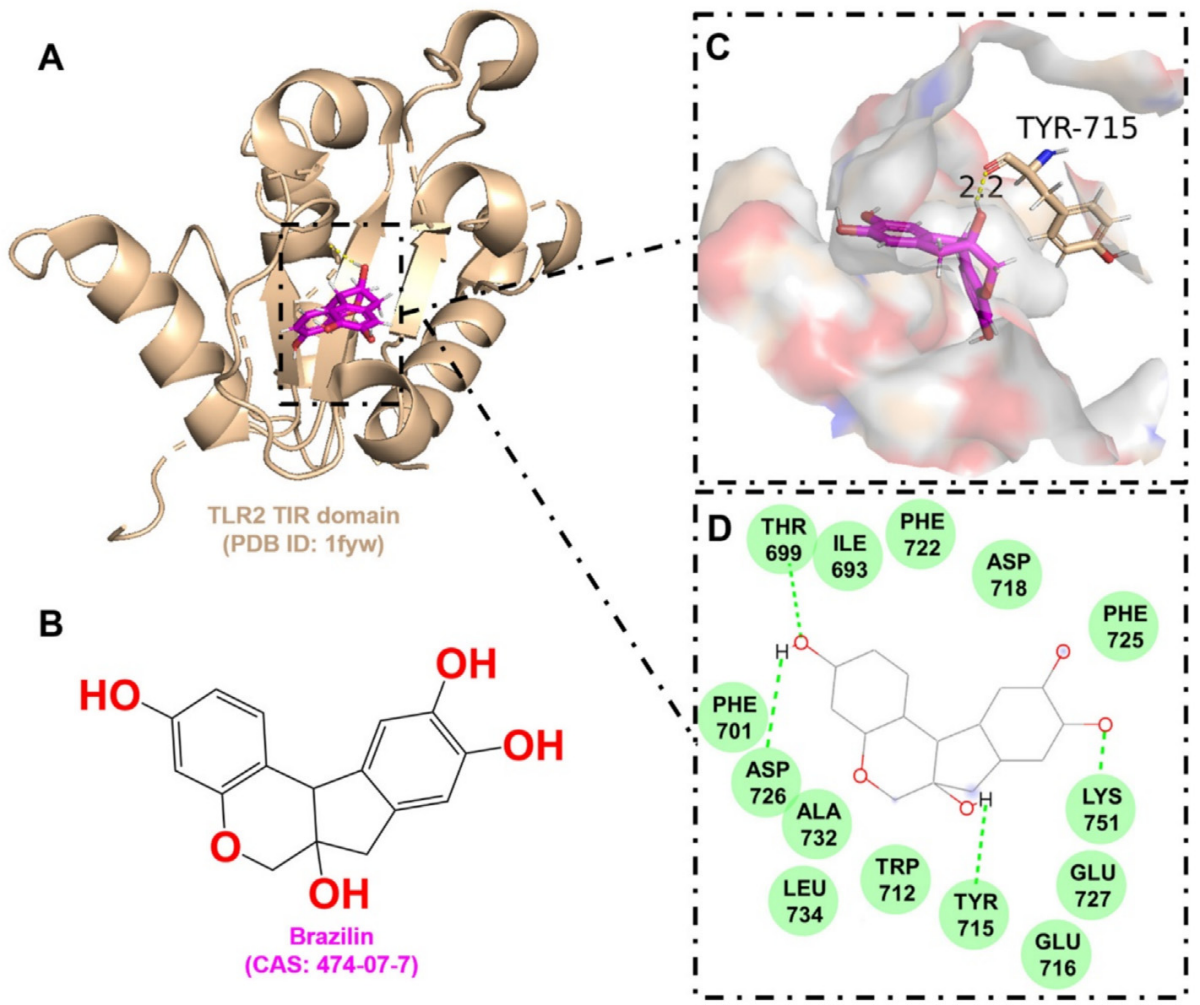
The interaction between brazilin and the TLR2 TIR domain. A. The 3D cartoon model of overall structure of the TLR2 TIR domain (PDB ID: 1fyw). B. The chemical formula of brazilin (CAS: 474–07-7). C. The close-up view of the interaction between brazilin and the TLR2 TIR domain. The residues involved in the binding are shown in wheat (stick model) with their names labelled, while the ligand is shown in magenta. The hydrogen bonds are drawn as yellow dashed lines, with distances labeled. The images were generated using PyMOL software. D. The 2D interaction postures were also generated with van der Waals interactions shown in green. (For interpretation of the references to colour in this figure legend, the reader is referred to the web version of this article.)

Currently, no TLR2 inhibitors have been approved for human use, making the study of the TLR2 binding pocket an important area of research [172]. The activation of TLR signaling is triggered by the dimerization of the intracellular TIR domains [173,174]. This process facilitates the recruitment of the adapter protein MyD88 to the TIR domains, initiating downstream signaling pathways that lead to the production of proinflammatory cytokines (Fig. 3). Therefore, inhibiting TLR TIR domain dimerization may provide a strategy to mitigate TLR2-driven hyperinflammation. Mistry and colleagues have utilized computational techniques to highlight the critical role of the BB-loop pocket residues in TLR2 TIR domains, suggesting that these residues (TYR647, CYS673, ASP678, PHE679, ILE680, LYS683, ASP687, ASN688, ASP691, and SER692) could be targeted for therapeutic intervention [175]. In the docking of TLR2 with Brazilin, it was observed that the active pocket is located within the CD-loop region of the TIR domain, which is also crucial for TLR signaling (Fig. 6). The CD-loop region has been identified as crucial for the dimerization and interaction of the TIR domains in TLR2, TLR4, TIR10, and IL-1RAcP, as highlighted in several studies [[176], [177], [178]]. It is among the most structurally variable regions of the TIR regions, with low sequence conservation. This variability likely facilitates the specific recognition and targeting of host TIR domain-containing proteins by microbial partners [179].

**Fig. 7:**
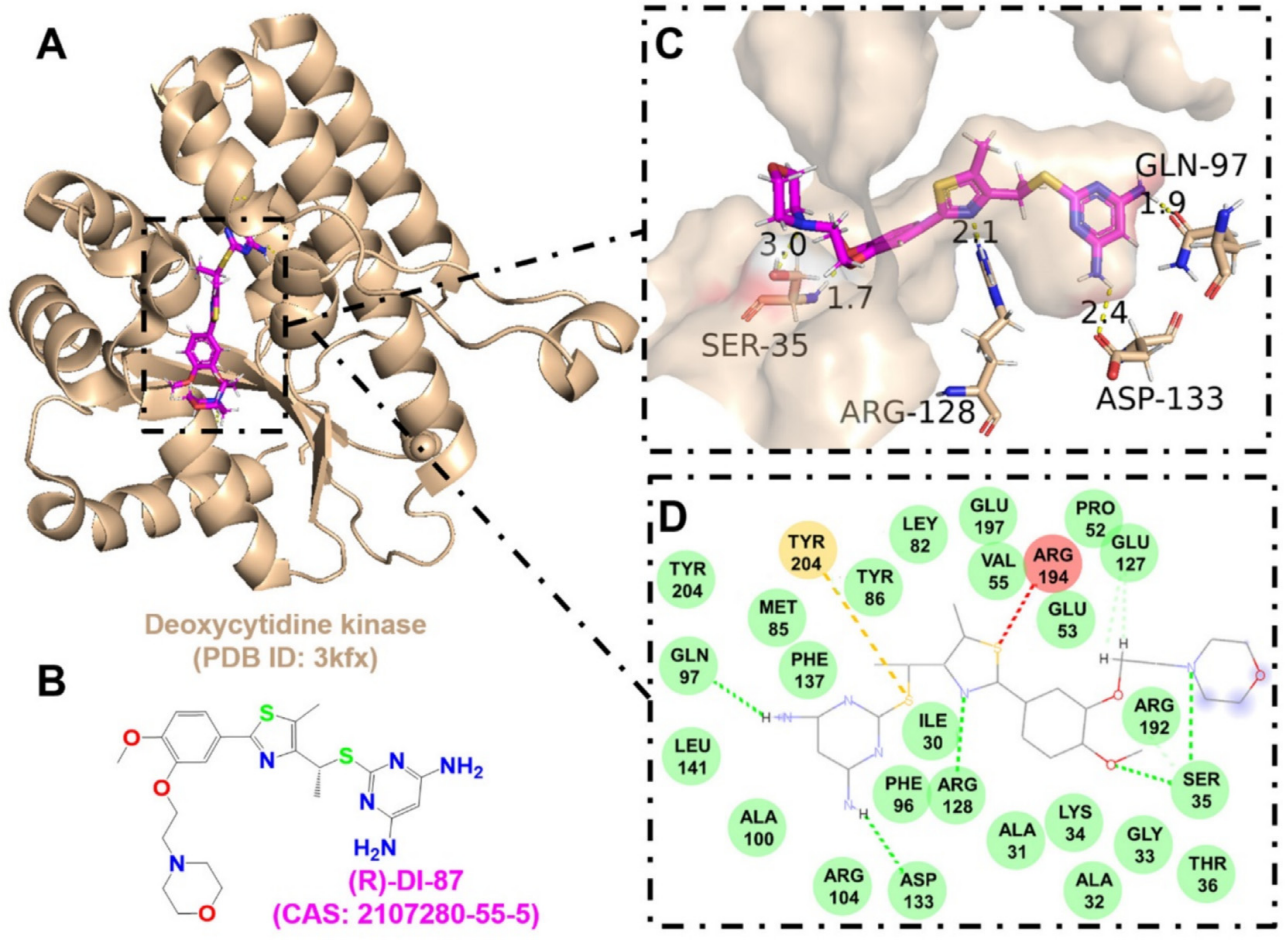
The interaction between (R)-DI-87 and deoxycytidine kinase. A. The 3D cartoon model of overall structure of the deoxycytidine kinase (PDB ID: 3kfx). B. The chemical formula of (R)-DI-87 (CAS: 2107280–55-5). C. The close-up view of the interaction between (R)-DI-87 and deoxycytidine kinase. The residues involved in the binding are shown in wheat (stick model) with their names labelled, while the ligand is shown in magenta. The hydrogen bonds are drawn as yellow dashed lines, with distances labeled. The images were generated using PyMOL software. D. The 2D interaction postures were also generated with van der Waals interactions, pi-sulfur, and unfavourable bump shown in green, yellow, and red dashed lines, respectively. (For interpretation of the references to colour in this figure legend, the reader is referred to the web version of this article.)

**Fig. 8:**
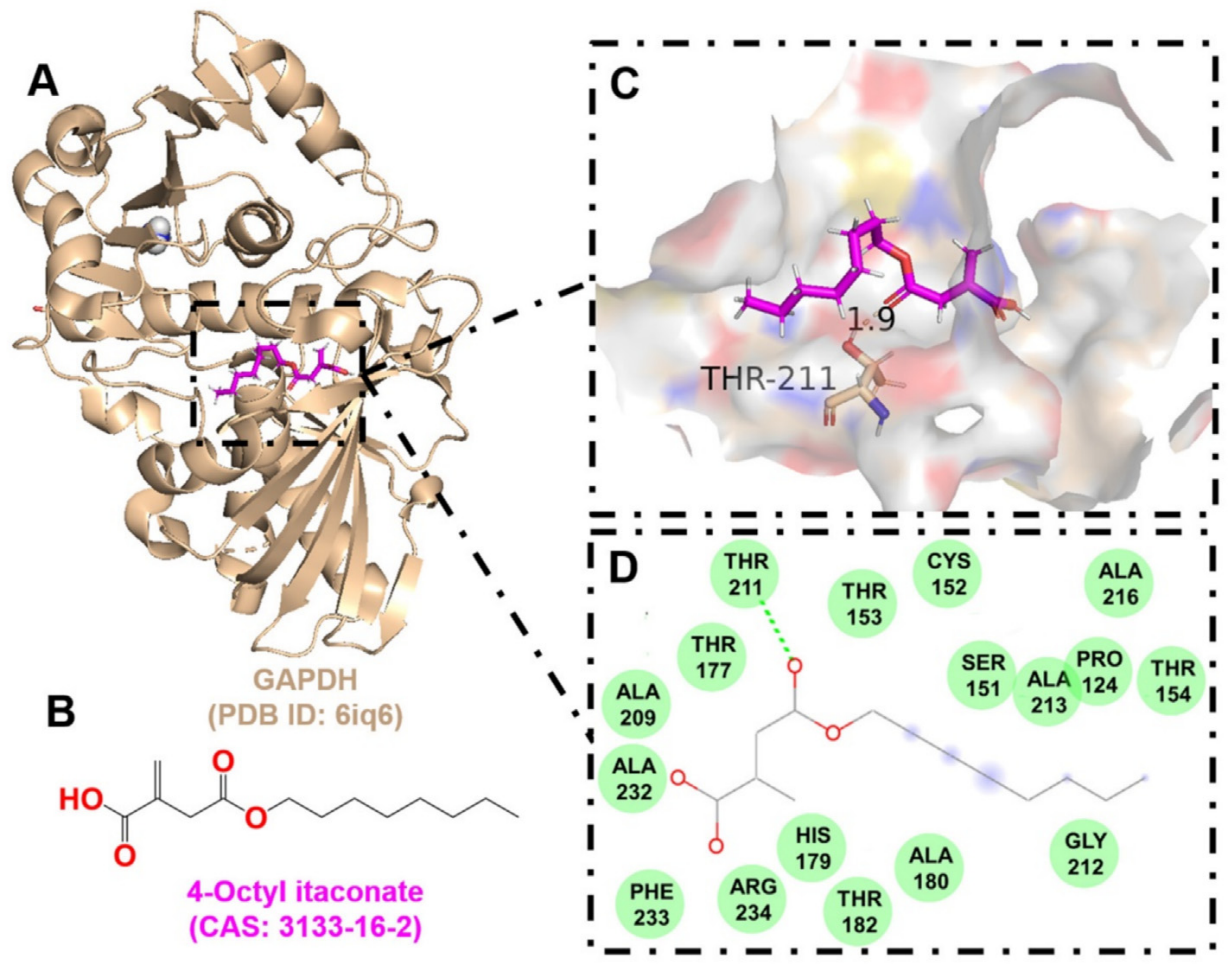
The interaction between 4-octyl itaconate and GAPDH. A. The 3D cartoon model of overall structure of GAPDH (PDB ID: 6iq6). B. The chemical formula of 4-octyl itaconate (CAS: 3133–16-2). C. The close-up view of the interaction between 4-octyl itaconate and GAPDH. The residues involved in the binding are shown in wheat (stick model) with their names labelled, while the ligand is shown in magenta. The hydrogen bonds are drawn as yellow dashed lines, with distances labeled. The images were generated using PyMOL software. D. The 2D interaction postures were also generated with van der Waals interactions shown in green. (For interpretation of the references to colour in this figure legend, the reader is referred to the web version of this article.)

**Fig. 9:**
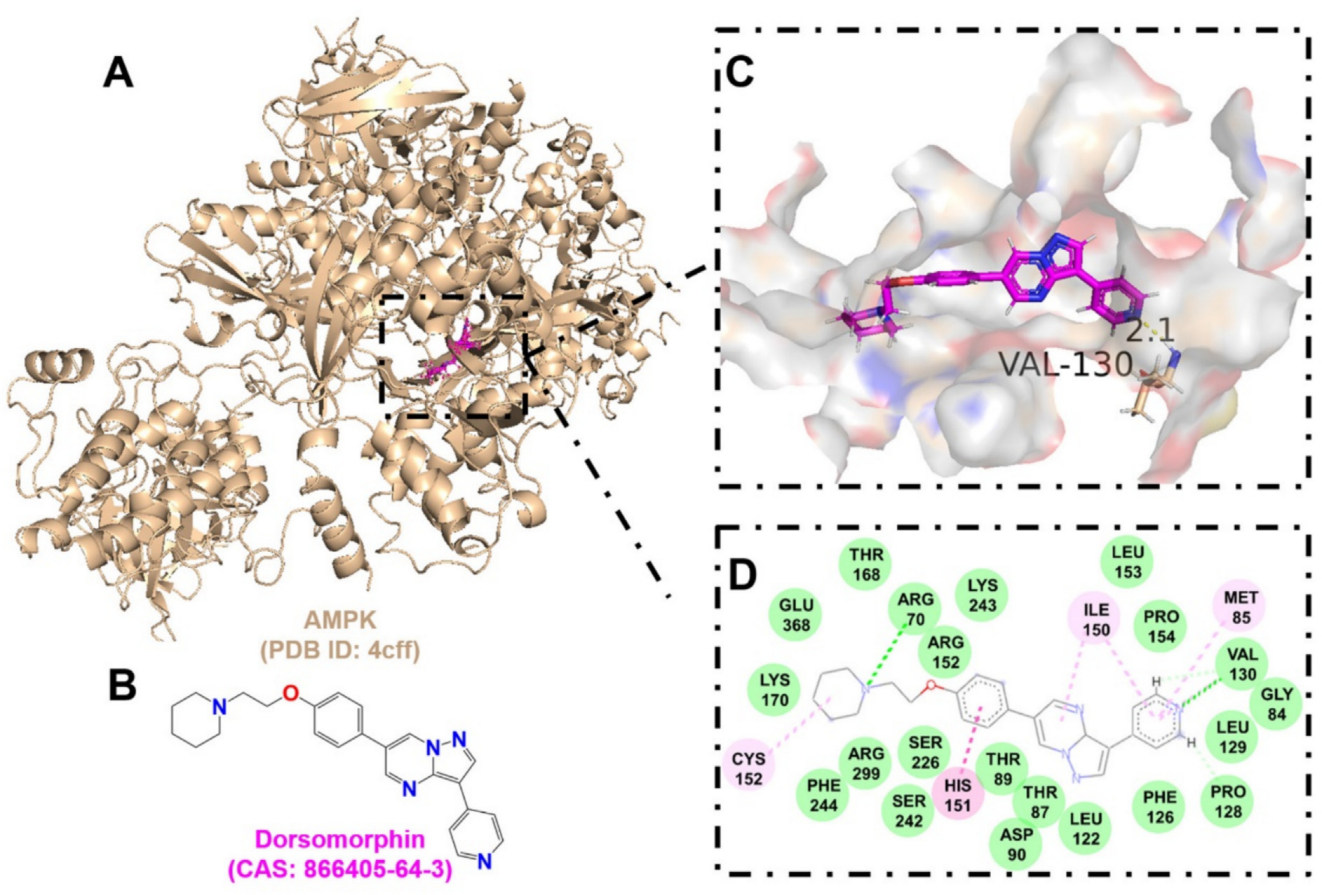
The interaction between dorsomorphin and AMPK. A. The 3D cartoon model of overall structure of AMPK (PDB ID: 4cff). B. The chemical formula of dorsomorphin (CAS: 866405–64-3). C. The close-up view of the interaction between dorsomorphin and AMPK. The residues involved in the binding are shown in wheat (stick model) with their names labelled, while the ligand is shown in magenta. The hydrogen bonds are drawn as yellow dashed lines, with distances labeled. The images were generated using PyMOL software. D. The 2D interaction postures were also generated with van der Waals interactions, carbon hydrogen bond, pi-alkyl, and pi-pi stacked shown in green, light green, pink, and light red dashed lines, respectively. (For interpretation of the references to colour in this figure legend, the reader is referred to the web version of this article.)

**Fig. 10:**
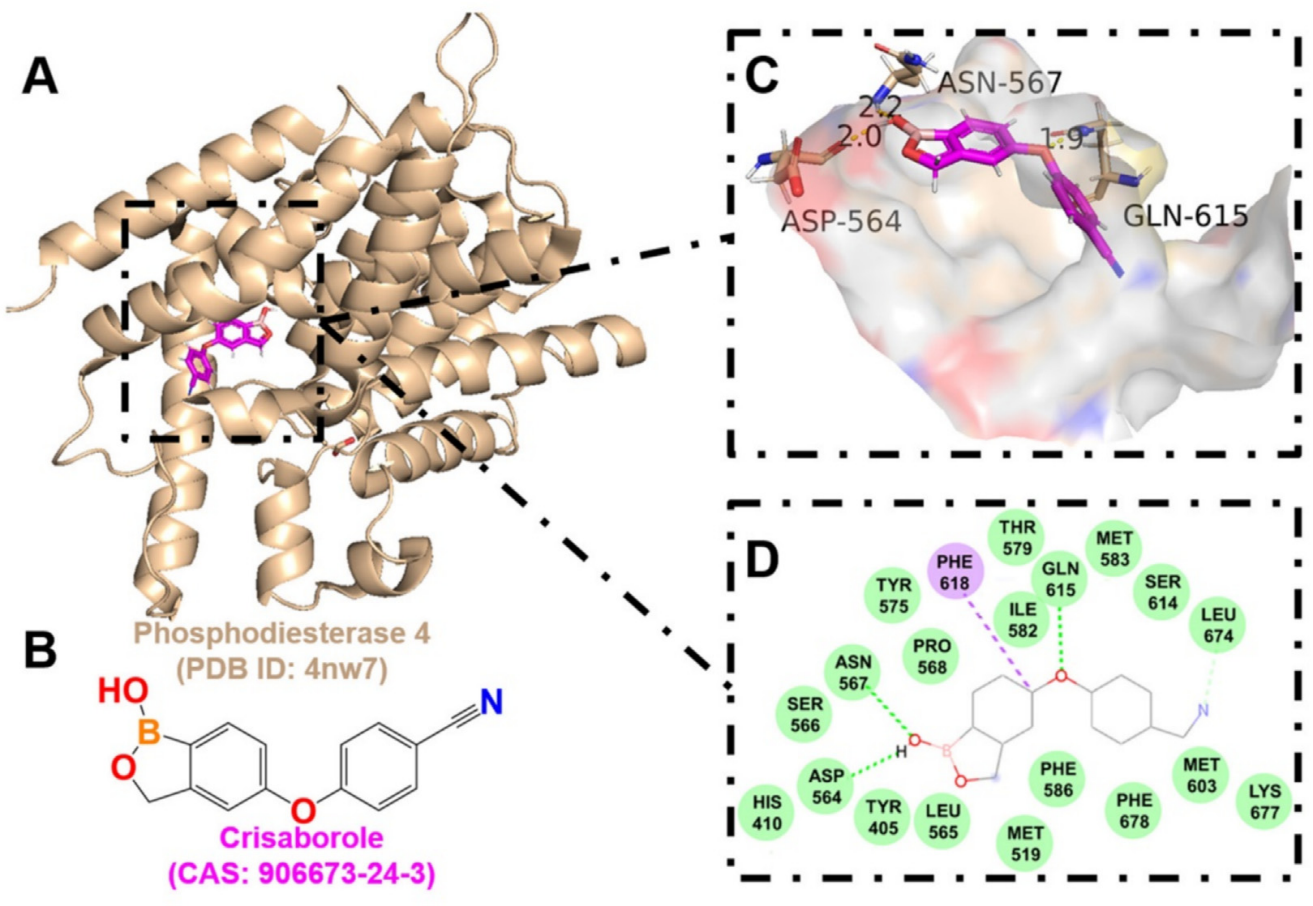
The interaction between crisaborole and phosphodiesterase 4. A. The 3D cartoon model of overall structure of phosphodiesterase 4 (PDB ID: 4nw7). B. The chemical formula of crisaborole (CAS: 906673–24-3). C. The close-up view of the interaction between crisaborole and phosphodiesterase 4. The residues involved in the binding are shown in wheat (stick model) with their names labelled, while the ligand is shown in magenta. The hydrogen bonds are drawn as yellow dashed lines, with distances labeled. The images were generated using PyMOL software. D. The 2D interaction postures were also generated with van der Waals interactions and pi-sigma shown in green and purple dashed lines, respectively. (For interpretation of the references to colour in this figure legend, the reader is referred to the web version of this article.)

Furthermore, to provide valuable insights for the design of novel antimicrobial drugs targeting the host, the active binding pockets of deoxycytidine kinase (Fig. 7), GAPDH (Fig. 8), AMPK (Fig. 9), and phosphodiesterase 4 (Fig. 10) were predicted using AutoDock Vina v1.2.x, and visualized using PyMOL 3.1. These target proteins were found to exhibit strong affinity for known HDADs. This suggests that targeting host proteins or pathways involved in immune response, oxidative stress, inflammation, skin barrier function, cell membrane integrity, and cellular autophagy holds significant potential for overcoming drug resistance and immune evasion in *S. aureus* infections.

## Conclusion and prospects

HDTs have recently attracted significant attention and been extensively studied for their ability to combat a wide range of pathogens, including bacteria [31,35,180], viruses [[181], [182], [183]], rickettsia [184], fungi [185], and parasites [186,187]. Among them, HDADs with broad-spectrum effects offer potential solutions to drug resistance and immune evasion, especially in managing unidentified infections, by rapidly modulating the host and reducing selective pressure on pathogens [31]. Despite extensive research, HDADs have not yet been translated into clinical applications. HDTs may also present challenges that warrant careful consideration in future studies.

### Limitations and considerations in HDT implementation

HDTs may be influenced by intracellular pathogens, thereby reducing the overall effectiveness of the therapy. Bacteria are evolving various defense mechanisms to counteract host response. *S. aureus* possesses pigmentation, such as staphyloxanthin [188] and carotenoids [189], which protect against ROS and confer resistance to phagocytic killing. Detoxifying enzymes like globins, peroxiredoxins, catalases, and superoxide dismutases prevent the accumulation of O^−^_2_ [190]. Metal homeostasis systems tightly regulate the transport and storage of metal ions like copper, zinc, manganese, and iron to prevent ROS generation *via* Fenton chemistry [191]. DNA repair mechanisms, including excision repair and recombinational repair, maintain genetic integrity damaged by oxidation [115]. Protein repair systems, involving thioredoxin, coenzyme A reductase, and methionine sulfoxide reductase, repair damaged proteins [190]. Moreover, bacterial efflux pumps [192] and antioxidants [193] can undermine the enhanced antibacterial activity induced by HDADs. At the same time, intracellular bacteria may hijack host defense mechanisms, such as autophagy, to support their survival or persistence [194]. Staphylococcal superantigen-like proteins can disrupt TLR2 recognition by blocking ligand binding and heterodimer formation. Additionally, the TIR-containing protein TirS in *S. aureus* mimics the host TIR domain, thereby inhibiting TLR2-mediated NF-κB activation. Furthermore, the murine paired Ig-like receptor-B, which binds *S. aureus* lipoteichoic acid, also acts to negatively regulate inflammatory responses.

Host-targeted approaches can have distinct effects depending on the bacterial infection. For example, enhancing the acidification of host lysosomes can promote the clearance of *S. aureus* [93] and *Mycobacterium tuberculosis* [195], but it may stimulate intracellular *Salmonella typhimurium* virulence gene expression, which promotes *Salmonella*-containing vacuole (SCV) rupture. The rupture of SCV then facilitates the pathogen's access to the cytosol, leading to cytosolic escape [196]. Additionally, mito-TEMPO selectively interferes with the growth of rough-type *Mycobacterium abscessus* by disrupting the mtDNA-driven IFN-I signaling pathway, with no effect on smooth-type isolates [197].

The dosage and timing of HDTs require careful optimization, with particular attention to potential side effects. TLR2 is a promising therapeutic target for infectious diseases, inflammatory conditions, and allergic reactions. However, excessive activation of TLR2 can lead to a persistent inflammatory state, which may contribute to autoimmune and chronic inflammatory diseases [198]. Additionally, extensive research has been conducted on PDE4 inhibitors as potential therapies for a range of human diseases. Despite this, the development of several PDE4 inhibitors has been halted in clinical trials, largely because of their narrow therapeutic window and significant adverse effects, such as emesis, which arise from their insufficient selectivity among different PDE4 subfamilies [199]. HDTs may cause off-target effects that disrupt cellular functions, whereas genetic variability and health conditions in hosts can lead to inconsistent treatment outcomes, both hindering the development of universal therapies [31]. Addressing these challenges requires precision medicine, comprehensive preclinical/clinical studies, and systematically optimized HDT designs. Despite these limitations, further research into HDTs is essential. Current data on *S. aureus* HDTs remain limited to preclinical research; however, evidence from other bacterial infections—particularly tuberculosis—suggests their considerable potential for future clinical applications in treating bacterial infections [18,200,201].

### Future research priorities in *S. aureus* HDT development

There is currently a substantial body of research on HDTs targeting bacteria other than *S. aureus*. These potential targets and therapeutic agents should be prioritized in future studies on HDTs against *S. aureus*. For instance, antagonists targeting G protein-coupled receptors can disrupt calcium transport, which impairs the growth of *Brucella abortus*, *Rickettsia conorii*, *Legionella pneumophila*, and *Coxiella burnetiid*. In contrast, therapies targeting cholesterol trafficking significantly reduce the intracellular proliferation of *L. pneumophila* and *C. burnetiid* [184]. Ephrin type-A receptor 2 has been identified as a potential universal receptor mediating the internalization of various bacteria into host cells. Its involvement in bacterial infections has been implicated in several pathogens, including *E. coli* [33], *Chlamydia trachomatis* [202], and *M. tuberculosis* [203]. GABAergic signaling boosts host defense by inducing autophagy, promoting phagosome maturation, and regulating inflammation. This provides broad-spectrum antibacterial effects against intracellular pathogens like *Mycobacterium*, *Salmonella*, and *Listeria in vivo* [94]. Moreover, Shigella flexneri requires Bruton's tyrosine kinase (BTK)-mediated phosphorylation of N-WASP to promote actin-based dissemination and motility. Building on this insight, ibrutinib, a BTK inhibitor, has been investigated as a potential therapeutic strategy [204].

The discovery and development of HDADs have been greatly facilitated by breakthroughs in biotechnology and the integration of various multidisciplinary approaches. These include advanced techniques such as high-throughput sequencing [205,206], high-content imaging [207], organoid technology [208], RNA interference [157], CRISPR interference [209], cell sorting [210], computer-aided drug design [211,212], proteolysis-targeting chimeras [213,214], artificial intelligence [215], nanotechnology [110,216], and the repurposing of existing drugs [33,217]. HDADs hold significant promise for treating infections caused by intracellular pathogens, particularly in cases of persistent and recurrent infections where the causative bacteria remain unidentified. When used in combination with antibacterial agents, HDADs are strongly recommended for the effective elimination of pathogenic bacteria in clinical settings. For instance, in tuberculosis treatment, statins as HDTs enhance autophagy in macrophages to improve the bactericidal efficacy of first-line antibiotics like rifampin and pyrazinamide. Similarly, in sepsis, IL-7 therapy restores T cell function, synergizing with antibiotics to combat bacterial infections [18]. However, such combination strategies have not yet been reported in clinical applications for *S. aureus* infections. It should be noted that the current findings primarily advance the fundamental understanding of HTDs against *S. aureus* infections. Further translational studies will be required to bridge these mechanistic insights into clinical applications.

## Consent for publication

The authors have consented to publish this article.

## CRediT authorship contribution statement

**Youle Zheng:** Writing – review & editing, Writing – original draft, Visualization. **Jin Feng:** Writing – review & editing. **Qianwei Qu:** Writing – review & editing. **Yongzheng Liu:** Writing – review & editing. **Yadan Zheng:** Writing – review & editing. **Yanhua Li:** Writing – review & editing, Funding acquisition, Conceptualization.

## Ethics approval and consent to participate

Not applicable.

## Funding

This work was supported by 10.13039/501100001809National Natural Science Foundation of China U24A20452.

## Declaration of competing interest

The authors declare that they have no known competing financial interests or personal relationships that could have appeared to influence the work reported in this paper.
